# Temporal Trends of Legacy and Current-Use Halogenated Flame Retardants in Lake Ontario in Relation to Atmospheric Loadings, Sources, and Environmental Fate

**DOI:** 10.1021/acs.est.3c04876

**Published:** 2023-09-11

**Authors:** Wen-Long Li, Tana V. McDaniel, Shane R. de Solla, Lisa Bradley, Alice Dove, Daryl McGoldrick, Paul Helm, Hayley Hung

**Affiliations:** † Air Quality Processes Research Section, Environment and Climate Change Canada, 4905 Dufferin Street, Toronto, Ontario M3H 5T4, Canada; ‡ Water Quality Monitoring and Surveillance Division, Environment and Climate Change Canada, Burlington, Ontario L7S 1A1, Canada; § Ecotoxicology and Wildlife Health Division, Environment and Climate Change Canada, Burlington, Ontario L7S 1A1, Canada; ∥ Environmental Monitoring and Reporting Branch, Ontario Ministry of the Environment, Conservation and Parks, 125 Resources Road, Toronto, Ontario M9P 3V6, Canada

**Keywords:** flame retardants, Great Lakes, multimedia, fate modeling

## Abstract

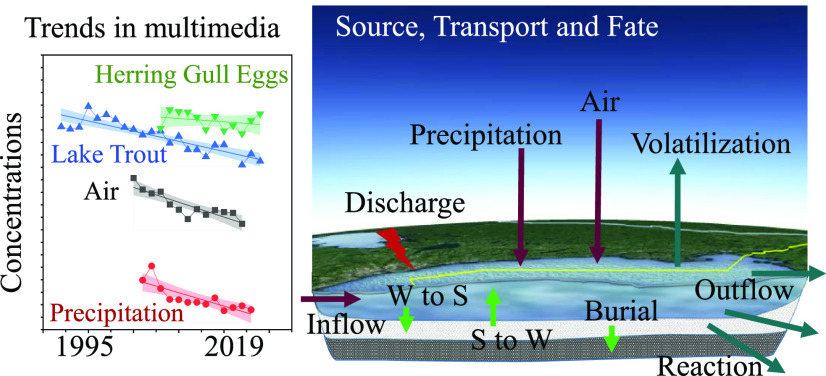

Since the phase-out of polybrominated diphenyl ethers (PBDEs), large amounts of alternative halogenated flame retardants (AHFRs) have been introduced to the market. Due to their persistence and toxicity, halogenated flame retardants (HFRs) have become a concern for the ecosystem and human health. However, there remains limited assessment of the atmospheric loadings, sources, and environmental fate of HFRs in Lake Ontario, which receives urban-related inputs and cumulative chemical inputs from the upstream Great Lakes from Canada and the United States. We combined long-term measurements with a modified multimedia model based on site-specific environmental parameters from Lake Ontario to understand the trends and fate of HFRs. All HFRs were detected in the air, precipitation, lake trout, and herring gull egg samples throughout the sampling periods. General decreasing trends were found for PBDEs, while the temporal trends for AHFRs were not clear. Physical–chemical properties and emissions significantly influence the levels, profiles, and trends. Using the probabilistic modeling, HFR concentrations in lake water and sediment were predicted to be close to the measurement, suggesting a good performance for the modified model. The loadings from tributaries and wastewater effluent were the primary input pathways. Transformations in the water and sedimentation were estimated to be the dominant output pathway for the three HFRs.

## Abstract 2

Temporal trends and source/fate modeling of HFRs in Lake Ontario reveal increasing levels and transportation fluxes of alternative HFRs, highlighting the potential threat to human health and aquatic organisms.

## Introduction

A subset of halogenated flame retardants (HFRs) was listed as prioritized substances by the Canadian Government’s Chemicals Management Plan (CMP) in 2006, operating under the Canadian Environmental Protection Act (CEPA), 1999, to protect the environment and the health of Canadians.^1,2^ As the traditional and the most widely used HFRs, polybrominated diphenyl ethers (PBDEs) were added to the Stockholm Convention and subsequently were mostly removed from the global market. After the regulations restricting the production and importation of PBDEs, alternative HFRs (AHFRs) were marketed as replacements for PBDEs with high demand to reduce the flammability of commercial products. For instance, global production of 1,2-bis(2,4,6-tribromophenoxy)ethane (BTBPE) and decabromodiphenylethane (DBDPE) increased in response to the restrictions of commercial decaBDE and octaBDE formulations, respectively.^3−5^ Commercial Firemaster 550 which contains 2-ethylhexyl-2,3,4,5-tetrabromo-benzoate (EHTBB) was used to replace the commercial pentaBDE mixture.^6^ First used in the 1960s, dechlorane plus (DPs or DDC-COs) were marketed as a replacement for the commercial decaBDE.^7,8^ Recently, these AHFRs have been receiving intensive public attention because of the elevated levels in the environment and their potential for persistence, bioaccumulation, and toxicity.^9−16^ For example, EHTBB has been suggested to be hazardous to the environment and humans^17,18^ and negatively affect exposed chicken embryos.^19^ DBDPE was reported to potentially biomagnify among fish in the food web of Lake Winnipeg (Canada).^20^

It is essential to understand the sources and fates of these HFRs as they have been frequently detected in the Great Lakes Basin (GLB), the most extensive freshwater system in the world. The status of pollution in the GLB is a priority concern for the United States (US) and Canada, with efforts being made by both sides to monitor pollution across different environmental components to evaluate the effectiveness of mitigation measures.^21−23^ As the lake furthest downstream in the Great Lakes, Lake Ontario (ON) reflects the cumulative chemical inputs and outputs of all lakes and major rivers upstream, from both Canada and the US. The water quality of Lake ON is thus affected by the cumulative input of industrial chemicals from upstream human activity. On the Canadian side, Lake ON is the industrial and population center of Canadian cities such as Hamilton and Toronto.^24^

Air and precipitation are important sources of anthropogenic chemicals that affect the health of the ecosystem in the GLB. There can be significant loadings of persistent pollutants in lake water from atmospheric deposition, some of which may bioaccumulate in the food web, potentially causing harm to both humans and wildlife living in the GLB. Therefore, the study of the HFRs in the GLB can provide valuable scientific information that helps us develop scientific strategies to control both emissions and thus loadings into the GLB.^25^ Appropriate modeling of the environmental fate of FRs is critical for developing effective control strategies for the chemicals.^26^

In this study, the levels and temporal trends of HFRs were investigated in the air, precipitation, lake trout, and herring gull eggs from Lake Ontario. The measured concentrations in the GLB were combined with a modified quantitative water air sediment interaction (QWASI) model using specific parameters, such as precipitation rate and water volume for Lake Ontario. The mass balance of the model was established to identify the most important input and output pathways. The modeled concentrations were compared with measurement concentrations in Lake Ontario to evaluate the performance of the model. Lastly, we determined if the temporal trends and fate of HFRs in both biotic and abiotic matrices mirrored those in the atmosphere in Lake Ontario. Our research will advance our understanding of the atmospheric processes of HFRs and their potential impacts on ecosystems.

## Materials and Methods

### Sampling and Sample Analysis

Details on the sampling and analyses of air and precipitation are summarized in the Supporting Information (SI) and previous studies.^27−29^ Briefly, A total of 263 pairs of gas- and particle-phase samples were taken at Point Petre (43.839 N, 77.152 W, see Figure S1) on Lake Ontario during 2005 and 2018, which is a regionally representative remote master station. A total of 157 precipitation samples were taken from 2006 to 2018 at the Point Petre site. Lake trout (*Salvelinus namaycush*) are collected for the analysis of contaminants in the Great Lakes and across Canada as part of the ECCCs Fish Contaminant Monitoring and Surveillance Program (FCMSP) to support the Chemicals Management Plan (CMP).^30^ A total of 243 fish samples were analyzed for HFRs from three sites close to the Point Petre site in the western, and central basin of Lake Ontario from 1997 to 2018. Herring Gull eggs have been collected annually as part of the Great Lakes Herring Gull Monitoring Program from 15 Herring Gull colonies throughout the Great Lakes and connecting channels since 1974.^31^ For this study, pooled herring gull samples from Snake Island (44.1908 N, 76.5432 W) which is close to Point Petre were included. The eggs were pooled in groups of 10–13 eggs per pool, and this pooling was conducted annually.

For air, the gas-phase and particle-phase samples were extracted by the Soxhlet and a Dionex ASE200 system, respectively. The precipitation sample was extracted twice using fresh dichloromethane. Whole fish samples from all years were analyzed by commercial laboratory SGS/AXYS (Sidney, British Columbia, Canada) using method MLA-033 equivalent to EPA Method 1614A as described in a previous study.^32^ Herring gull egg sample extraction and instrumental analysis of BFRs in gull eggs are described in detail in a previous study.^33^

### QA/AC and Trend Analysis

Detailed QA/QC, trend analysis by the Digital Filtration (DF) method, and potential source contribution function (PSCF) modeling can be found in the SI.

### Probabilistic Fugacity Modeling

The Quantitative Water Air Sediment Interaction (QWASI) model is a mathematical model used to simulate the transport and fate of contaminants in aquatic systems.^34−36^ QWASI is designed to consider the interactions between water, air, and sediment in a system, allowing for the prediction of the movement and behavior of contaminants in these different phases. The models were compiled using the fugacity concept and have been made freely available as software from the Canadian Centre for Environmental Modelling and Chemistry (CEMC) website and have been applied to specific environmental systems.^37^ Chemical concentration in the precipitation was predicted from air measurement in the original model. As we have measured precipitation concentration in this study, the original model can therefore be modified to incorporate the precipitation data and then applied to assess the fate of selected HFRs in Lake Ontario. Inputs of the QWASI model include the chemical properties^38^ (Tables S1 and S2) and the environmental parameters such as direct emissions (from wastewater treatment plants (WWTPs) and tributary runoff,^39^ details in the SI), inflows water, wet and dry atmospheric deposition, and lake-specific parameters. The mass balance of water and sediment compartments was established by steady-state equations. The model was run based on annual input of measured concentrations in the air, precipitation, river, and inputs from tributaries and WWTPs.

Additionally, the input parameters for the QWASI model were simulated using the Monte Carlo simulation technique 10 000 times. This approach allows for the generation of multiple plausible scenarios by sampling from probability distributions associated with each parameter. By incorporating Monte Carlo simulation, the QWASI model accounts for the inherent uncertainty and variability in the input parameters, providing a more comprehensive assessment of the transport and fate of HFRs in Lake Ontario.

## Results and Discussion

### HFRs in Air, Precipitation, Lake Trout, and Herring Gull Eggs

#### Air

The most frequently detected HFRs were BDE47, BDE99, BDE100, BDE209, and DPs with their detection frequencies ranging between 52 and 99% (Table S3). BDE47, BDE99, and BDE209 were the dominant PBDE congeners with an average concentration of 3.69 ± 4.77, 2.06 ± 5.3, and 2.3 ± 6.5 pg/m^3^, respectively (Figure S2). The concentrations of intermediate congeners (including BDE183, BDE196, BDE197, BDE202, and BDE203), the main components of the technical octaBDE mixture, were lower than the levels of lower-brominated congeners and BDE209. Lower-brominated congeners such as BDE17, BDE28, BDE47, and BDE99 are found mainly in the technical pentaBDE mixture. For AHFRs, the concentrations of DBDPE, EHTBB, HBCDs (sum of three isomers), and DPs were close to that of BDE47, BDE99, and BDE209. The levels and detection frequencies of other targeted BFRs (ATE, BTBPE, DPTE, PBT, and PBE) were low. Average concentrations of HFRs were close to those of Eagle Harbor but much lower than those of Chicago.^40^ For example, BDE47 concentrations in air samples collected in 2005–2009 at the US Great Lakes sites were 5.0–15 pg/m^3^ at rural and remote locations.^40^ DecaBDE are still being used in large, albeit declining, quantities worldwide and consequently are present at higher levels in the atmosphere.

#### Precipitation

The most frequently detected PBDEs were BDE47, BDE99, BDE100, and BDE209 in the precipitation (Table S3), which is similar to those found in air, suggesting a similar atmospheric source and environmental behavior of PBDEs in air and precipitation. As the main components of the most heavily used PBDE commercial mixtures, BDE47, BDE99, and BDE209 were the dominant HFRs in precipitation with concentrations of 0.075 ± 0.16, 0.11 ± 0.20, and 0.61 ± 1.5 ng/L, respectively. The levels of BDE47, BDE99, and BDE209 detected in this study are in the same range as those measured in precipitation samples collected at the US Great Lakes sites.^41^ For nonPBDE HFRs, BTBPE, DBDPE, and HBCD were the dominant HFRs in precipitation with concentrations of 0.41 ± 0.28, 1.3 ± 2.3, and 2.0 ± 4.8 ng/L, respectively.

#### Lake Trout

BDE47, BDE99, BDE100, BDE153, and BDE154 congeners were the dominant congeners constituting ≥80% of the ∑PBDE concentrations of the lake trout from Lake Ontario. Average wet weight-based concentrations of BDE47, BDE99, BDE100, BDE153, and BDE154 were 40 ± 31, 11 ± 6.8, 9.8 ± 5.4, 3.5 ± 2.0, and 5.2 ± 2.6 ng/g, respectively. The pattern of PBDE congeners was generally comparable with previous studies in fish in the same area.^42^ Unlike abiotic media such as air and precipitation, BDE209 was not a dominant congener in biotic media likely due to its reduced bioavailability and tendency for biotransformation to lower-brominated PBDEs. Similar to BDE209, DBDPE undergoes debromination and thus contributes to the formation of lower-brominated DPEs in the environment.^43^ Relatively greater accumulation of BDE47 compared to BDE99 could result from various factors, including trophic magnification factors, uptake rate, and possibly metabolism. Previous studies have demonstrated that the lower-brominated BDE congeners generally have greater trophic magnification factors and that smaller fish generally have higher uptake rates.^44^

For nonPBDE HFRs, HBCDs were the dominant compound with an average concentration of 4.7 ± 2.8 ng/g, followed by DPTE with values of 3.1 ± 1.1 ng/g. HBCDs were found in Lake Trout and Walleye from the Great Lakes with the concentration ranging from 0.49 to 14 ng/g.^45^ Used as an additive FR for over 30 years, HBCDs are primarily used in polystyrene foam insulation in the building industry and to a lesser extent textile coatings.^46^ The sources of HBCDs and DPTE were inconsistent with other BFRs,^47^ which may contribute to the observed inconsistent trends. HBCD has been frequently detected in Great Lakes fish samples, consistent with other studies.^48^ DBDPE is an additive FR and, starting in the early 1990s was used as an FR replacement for the decaBDE. As an alternative chemical of BDE209, the concentration of DBDPE (0.49 ± 1.2 ng/g) was close to that of BDE209 (0.26 ± 0.45 ng/g).

#### Herring Gull Eggs

Similar to lake trout, BDE47, BDE99, BDE100, BDE153, and BDE154 congeners were the dominant congeners constituting ≥80% of the ∑PBDE concentrations of herring gull eggs from Lake Ontario (Table S4). Average wet weight-based concentrations of BDE47, BDE99, BDE100, BDE153, BDE154, and BDE209 were 83 ± 27, 82 ± 29, 41 ± 11, 36 ± 14, 22 ± 7.2, and 9.3 ± 9.1 ng/g, respectively. The detection frequency of these congeners ranged from 92% for BDE209 and 75 to 100% (average 94%) for other PBDEs. HBCD was the dominant nonPBDE HFR detected in 91.7% of the eggs with a concentration of 12 ± 16 ng/g.

### Temporal Trends

#### Air

BDE99, BDE153, and BDE154 showed a faster rate of decline at Point Petre with half-lives of 2.9–5.5 years than that of 12 years for BDE209 (Figure S3). BDE209 levels were decreasing slowly at Point Petre since there are still existing sources in urban areas. Point Petre is closer to urban areas, probably reflecting the replacement of the BDE technical mixtures in cities like Toronto. Manufactured items containing decaBDE are still permitted to be imported into Canada as of 2023, and Canada has proposed to prohibit the import and sale of manufactured items containing decaBDE, with time-limited exemptions.^49^ The half-life of lower-molecular-mass PBDEs was generally longer than that of higher-mass PBDEs, for example, BDE28 with a half-life of 25 years, which could be due to the continued inputs of atmospheric debromination from higher-brominated PBDEs.^50^ Ma et al.^13^ reported a decreasing trend for atmospheric BDE209 with a half-life of 5 years at Cleveland and Sturgeon Point between 2005 and 2011. Venier and Hites^51^ and Salamova and Hites^40^ reported fast declines in PBDEs, with a half-life of 3.4–4.0 years and 6.3 years at the US IADN sites between 2005 and 2006 and 2005 and 2009, respectively. Liu et al.^52^ reported declining trends of PBDEs at two urban sites from 2005 to 2013.

The temporal trends for AHFRs are not as straightforward as those for PBDEs. The absence of clear temporal effects suggests continued inputs of AHFRs into the environment. PBT concentrations are increasing at Point Petre, with a doubling time of 20 years. DBDPE also demonstrated a steep inclining trend between 2012 and 2017, but the incline leveled off in 2017 at Point Petre, with a doubling time of 2.6 years. The doubling time (1.3 years) of HBCD suggests a fast-increasing trend. Syn-DP and anti-DP levels are decreasing, with a half-life of 4.5 and 6.7 years, respectively, at Point Petre.

#### Precipitation

The concentrations of PBDEs in precipitation at PPT decreased with halving times ranging between 1.9 and 15 years, depending on the congener, similar to declines in precipitation reported for the US IADN sites during 2005–2009.^40^ Overall ∑PBDE concentrations are declining in the atmosphere of the Great Lakes with an average halving time of ∼6 years (Figure S4).

For AHFRs, due to low detection frequencies, only trends of PBEB and HBCD in precipitation could be derived. Concentrations of PBEB did not change significantly (Figure S4), consistent with findings reported in precipitation in the US Great Lakes during 2005–2009.^40^ Similarly, PBEB was identified in Canadian lake trout caught in 1979.^53^ More recent studies on PBEB are scarce and scattered. HBCD concentrations decreased from 2006 to 2011 but started to increase after 2011 both in air and precipitation. Increasing trends in HBCD in atmospheric media of the Great Lakes basin are concerning as it is a chemical of mutual concern and listed as a persistent organic pollutant (POP) under the Stockholm Convention.^54^

#### Lake Trout and Herring Gull Eggs

Figure S5 shows the temporal trends of HFRs in the lake trout. Of the lake trout sampled, median ages slightly increased from 5.0 years in 1997 to 6.0 years in 2019 (Table S5). However, no significant difference was evident across years in the median values of body size (total length, fork length, total weight), lipid content, and moisture content (*p* ≥ 0.05). Therefore, the time trends were not adjusted by these parameters. Concentrations of most HFRs in lake trout significantly decreased from 1996 to 2019. The halving time ranged from 6.1 years for BDE209 to 16 years for BDE154. This pattern differs slightly from a previous study which showed an increasing trend in BDE100, BDE153, and BDE154 between 2011 and 2016 in the western basin of the lake;^32^ that increasing trend appears not to have continued when the period was extended. Due to limited data available for most AHFRs, only the time trends of DBDPE, HBB, HBCDs, and PBEB were estimated. Su et al. found a significant decline in α and γ-HBCD in lake trout from 2008 to 2016 in Lake Ontario.^55^ For herring gull eggs (Figure S6), the halving time for BDE66 was 5.6 years (*p* < 0.05). The time trends for most of the target compounds were not significantly decreasing (*p* ≥ 0.05), which could be due to the limited amount of data points as egg samples were pooled into one sample for the same year. Using pooled samples could be a limitation when looking for finer-scale time trends due to potential drawbacks associated with analyzing grouped samples instead of individual specimens.^56^ The concentrations of HFRs in the environment are positively related to their production levels. In the US, some AHFRs such as PBEB and HBCDs were produced and used in the 1970s and 1980s. In 1986, 4.5–226.8 tonnes of PBEB were produced;^40^ however, there seems to be no current information on the US production of PBEB. HBCD production worldwide peaked in 2013 and then declined rapidly thereafter.^57^ Domestic production of HBCD in the US reportedly ceased by 2016, but imports of HBCD from China have increased substantially from 2014 to 2016 with a total of 383.8 tonnes in 2016.^58^

### Physical–Chemical Properties and Emissions Influence the Levels, Profiles, and Trends

#### Factors Governing Concentrations

Ambient temperature may be the most influential parameter of HFR concentrations in air and precipitation because it directly affects vapor pressure and octanol–air partition coefficients (*K*_OA_), and hence gas–particle partitioning. As shown in Figure S7, concentrations of lower-molecular-mass HFRs were significantly and positively correlated with air temperature, with correlation coefficients greater than 0.48 (*p* < 0.001). The influence of temperatures was also compound-specific. Compared to higher-molecular-mass HFRs, emissions of lower-molecular-mass HFRs such as BDE17, BDE28, BDE47, ATE, PBT, and PBEB were relatively more sensitive to temperature fluctuation. This is because HFR emissions increased with the elevated temperatures, and the slope of emission rates also rose sharply from consumer materials.^59^

The air–water partition coefficient (*K*_AW_) relates to gas-phase and aqueous-phase concentrations of a substance. Ambient temperature also plays an essential part because it directly affects *K*_AW_, hence and all related fate processes. Figure S8 shows significant but negative correlations between concentrations in the precipitation of many HFRs and the ambient temperature, with the correlation coefficients ranging between −0.60 (*p* < 0.001) and −0.19 (*p* < 0.05). This relationship indicated higher concentrations of HFRs in the precipitation during colder seasons. As *K*_AW_ increases with increasing temperature, lower temperatures in the colder season led to more HFRs partitioned to precipitation rather than air. Additionally, snow has a larger surface area for chemical adsorption and a higher scavenging ratio compared to rain.

Most of the HFRs in our study, such as PBDEs, are considered to be very persistent and bio-accumulative. The bioaccumulation factor (BAF) of HBB was higher than 5000 in aquatic biota (invertebrates, fish, and reptiles) from an e-waste site.^60^ Previous studies have shown that age is a significant factor influencing the levels of HFRs in fish.^42^ As shown in Figure S9, regressions of the HFR compound across years were used to determine which factors (e.g., fork length, weight, age, %lipid, and %moisture) were significantly related to the HFR concentrations in the lake trout. All of the factors showed a significant correlation with HFR concentrations in lake trout (*p* < 0.01). The negative relationship between %moisture and concentrations could be just coincidental. This is because, based on Table S5, the correlation between moisture and lipid was −0.73. HFRs are lipophilic and thus sequestered in lipids. Low moisture likely reflects high lipids (and vice versa), which are the main drivers of bioaccumulation. Positive influence (*p* < 0.01) on the fork length, weight, age, and %lipid accounted for the majority of variability in the HFR concentrations in lake trout. Body size, as estimated using fork length, was otherwise the most significant variable, which had significant correlations with the HFR concentrations with higher absolute values of correlation coefficients (|*r*| = 0.50–0.65) than other factors (|*r*| = 0.18–0.57). This study provides clear evidence that concentrations of most HFRs increase as a function of the age, body size, and lipid content of lake trout. The findings of this study strongly suggest that the age and body size of lake trout should be regarded as a critical factor in future HFR monitoring and surveillance of lake trout. No significant correlations with moisture or lipid content for the herring gull eggs were found.

#### Factors Governing Profiles

We expressed individual PBDEs congeners as a proportion of the sum PBDEs to examine congener profiles among matrices. The PBDE congener patterns in the air and precipitation were very similar, likely due to the partitioning and direct exchange of HFRs between air and precipitation as the rain falls through the atmosphere. More of the commercial decaBDE, which is relatively high in BDE209, was used in the 2000s relative to the other two technical mixtures (commercial pentaBDE and octaBDE).

However, the PBDE congener patterns in the lake trout and herring gull eggs differed from those in the atmosphere. This difference may be explained by differences in the bioavailability of the less-brominated congeners compared with the more-brominated congeners, as well as differential rates of metabolism and excretion of PBDE congeners. Generally, the rates of metabolism of POPs are the rate-limiting step in their elimination from body burdens;^61^ the capacity for metabolizing POPs is higher in endotherms like birds than it is in ectothermic vertebrates like fish.^62^ BAFs for BTBPE, TBB, and TBPH were generally an order of magnitude lower than those of BDE47, BDE99, BDE100, BDE153, and BDE154 (constituents of the commercial pentaBDE), indicating a lower potential for accumulation.^63^ The BAF values generally increase from BDE28 to BDE154 and subsequently decline from BDE154 to BDE209. In this study, the percentage of BDE209 in the lake trout and herring gull eggs was low compared to that in the atmosphere, due to the debromination and low bioavailability of BDE209.^64^ BAF for HBCDs is about two orders of magnitude lower than that of BDE47. However, significant concentrations of HBCDs can still be found in lake trout compared to other HFRs and could be due to higher loadings of HBCDs through the wet deposition.

#### Factors Governing Trends

Since the emissions of HFRs significantly influence their concentrations in the atmosphere, atmospheric trends of HFRs are related to the usage from nearby sources as well as from long-range atmospheric transport. Commercial pentaBDE and octaBDE were withdrawn from the market at the end of 2004, and the other major PBDE product, decaBDE, was withdrawn at the end of 2013.^52^ Other halogenated flame retardants have been introduced to replace these PBDE products. The decreasing time trends of PBDEs in the atmosphere mirror the phase-out of PBDEs and reflect historical changes in the chemicals used as flame retardants. Meanwhile, the increasing trends of many AHFRs suggest that these HFRs are still in use. Canada is the first jurisdiction to propose to ban DBDPE and products containing DBDPE and there is no indication of declining use of DBDPE.^49^ In 2016, the use of HBCDs was restricted in Canada,^65^ following the listing in the Stockholm Convention on Persistent Organic Pollutants (SC-POPs) in 2013; however, in the US, HBCDs are regulated in only a few states.^66^ On May 2023, DP was adopted to be listed in Annex A of the Stockholm Convention.^67^ Nonetheless, production of DP in the US putatively ceased in 2016, and China’s New Pollutant Management Action Plan indicated that the use of DP will be banned in China starting in 2026.^68^ Continuing measurements of the atmospheric concentrations of these and other chemicals used as flame retardants are warranted.

Concentrations of PBDEs in the Great Lakes environment have decreased in the past two decades, especially in the atmosphere^52^ and sediment.^69^ Both wet and dry deposition of most PBDEs to Lake Ontario has decreased based on the data in this study. Concentrations of most PBDEs have also declined in lake trout. Trends in HFRs in lake trout are influenced by changes in abiotic concentrations in the environment and their diet.

#### Linkage among the Four Environmental Media

**Figure 1:**
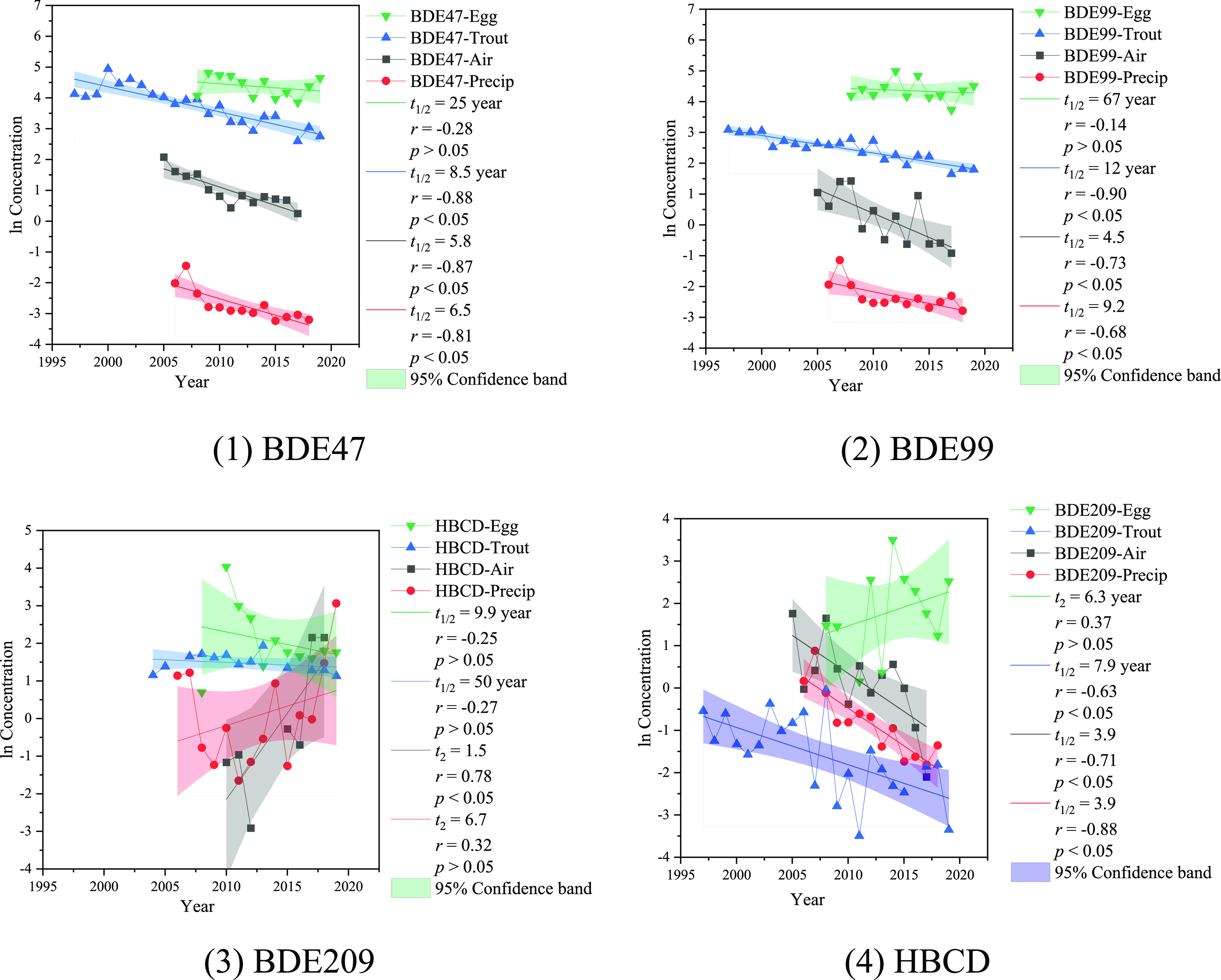
Comparisons on the natural logarithm of annual concentrations of PBDEs in the air (pg/m^3^), precipitation (ng/L), lake trout (ng/g), and herring gull eggs (ng/g).

HFR concentrations varied over time and location as well as among the environmental media in Lake Ontario. Generally, there were similar decreasing trends in HFRs in air, precipitation, and to some degree fish while concentrations in herring gull eggs showed little or no change across the study period; see Figure 1. The reasons for these differences are likely due to individual source-fate dynamics. Atmospheric concentrations of HFRs are directly impacted by the emissions from source regions while concentrations in biota are also impacted by bioavailability, metabolism and excretion rates, and changes in how contaminants move through the food chain.

Principal component analysis (PCA) was used to examine the relationship among the concentrations of HFRs in air, precipitation, lake trout, and herring gull eggs (see the SI). The pattern of HFRs was similar among both components for air and precipitation, whereas the distribution patterns of HFRs for fish samples were different from that of herring gull egg samples. BDE209, BDE47, BDE99, BDE100, and BDE183, which had the most influence on the PC1, were the most likely to be predominant in egg samples, distinguishing them from all other media. Compounds associated with PC2, BDE47, BDE99, BDE100, BDE49, BDE66, and HBCDs, tend to accumulate in biotic media. Bioaccumulation of BDE47, BDE99, BDE100, BDE49, and BDE66 is higher than other higher-brominated PBDEs.^64^ These findings emphasize the potential for utilizing these unique distribution patterns as ″specific fingerprints″ for source tracking purposes. By understanding the distribution patterns of specific HFR compounds across different media, we gain insights into their respective sources and potential accumulations within the ecosystem. This information could be pivotal for comprehending the origin and dynamics of HFR contamination.

### Source, Transportation, and Fate Modeling

The modified QWASI model was applied by using a probabilistic approach to investigate the transport and fate of three major HFRs, i.e., BDE47, BDE209, and HBCDs in Lake Ontario based on their high levels and detection frequency. In combination with the loadings from air and precipitation, the discharge data from WWTPs and tributaries in the Toronto region in 2008^39^ were used to predict concentrations of PBDEs in lake water. The predicted concentrations of BDE47 and BDE209 in lake water were 2.6 ± 1.2 and 3.0 ± 1.4 pg/L, respectively, which were underestimated by the factors of 13 and 5.7, respectively, compared to the measurements in 2011 with the concentrations of 34 ± 17 and 17 ± 11 pg/L.^70^

**Figure 2:**
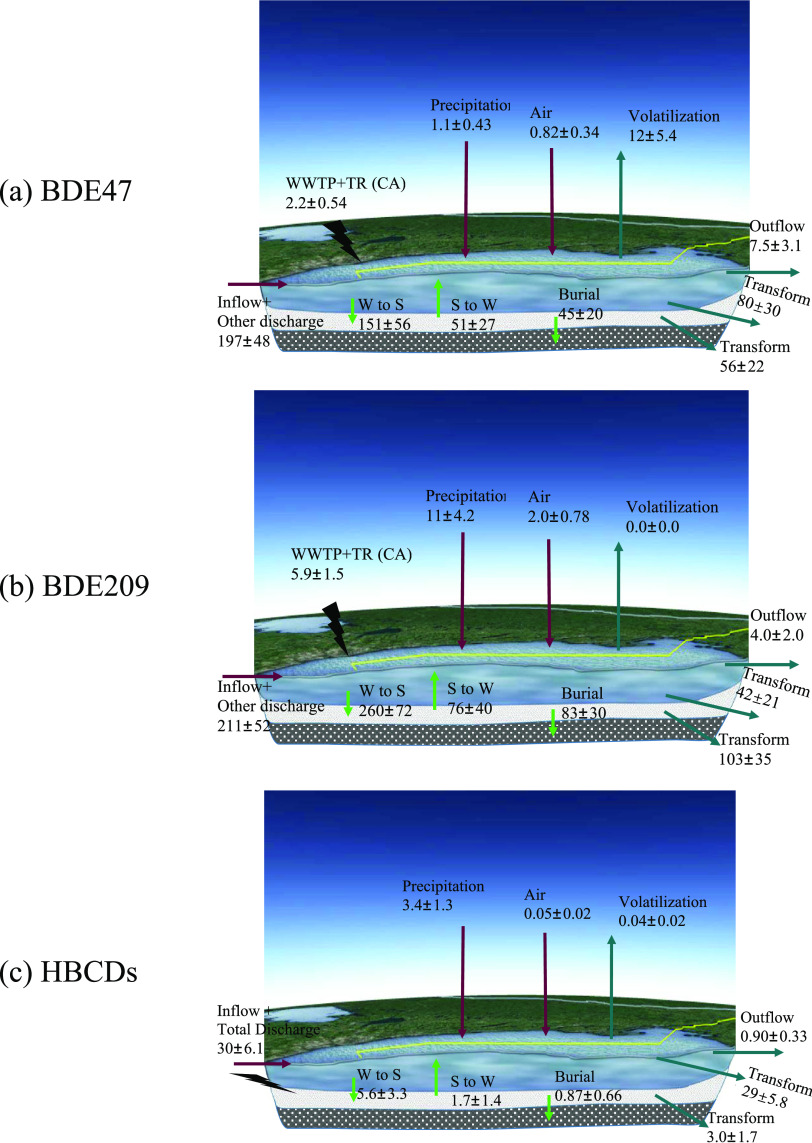
Mass balance of transportation flux (kg/year) for BDE47, BDE209, and HBCDs in Lake Ontario in 2011 by probabilistic modeling. Note: W and S represent water and sediment, respectively. Note that WWTP + TR (CA) represent WWTPs and tributaries from Canada.

To address this discrepancy, an inverse modeling method was employed to back-calculate the discharges from other potential sources, such as discharges from WWTPs and tributaries from the US side. Figure S10 shows the workflow for applying the modified QWASI and inverse modeling to estimate HFR emissions from the concentrations in water and sediment. This method has been used in previous studies to estimate the emissions of polycyclic aromatic hydrocarbons^71^ and organophosphate esters.^72^ Taking the modeling results from 2011 as an example (Figure 2), the transportation flux of input and output pathways for BDE47, BDE209, and HBCDs provide a systematic understanding of their environmental behavior in Lake Ontario. Mass balance was achieved with total inputs equal to the total output for each modeled compartment. For the input pathway, the loadings of BDE47 and BDE209 from tributaries and WWTP effluent from Canada were extrapolated as 2.1 ± 1.3 and 5.9 ± 3.3 kg/year, respectively, based on the halving times in the precipitation and the discharges in 2008.^39^ These values were higher than the loadings from precipitation and air, with values of 1.1 ± 0.43 and 0.82 ± 0.34 kg/year, respectively, indicating that the loading contributions from WWTPs and tributaries should not be ignored, consistent with loading pathway estimated for the Toronto area of Lake Ontario.^39^ Precipitation-to-water transfer showed greater contributions to total loadings than air-to-water transfer for BDE209 and HBCDs. Loadings from other discharges (inflow water plus the other sources from Canada and the US) were the dominant input pathway, with loadings of 197 ± 48 and 211 ± 52 kg/year for BDE47, and BDE209, respectively. These values for BDE209 were reasonable as a high annual production volume of 15,000 tons was reported from China in 2018 alone.^73^ As no discharge data from WWTPs and tributaries was available for HBCDs, the total discharges from all sources were calculated at 30 ± 6.1 kg/year in 2011. These results suggest that primary emissions from WWTPs and tributaries plus the water inflow were the dominant loadings to Lake Ontario for HFRs, while the atmospheric loadings played a secondary role.

For the output pathway, total transformations in the water and sediment were the dominant output pathway for the three HFRs, with values of 136 ± 36, 145 ± 42, and 32 ± 6.0 kg/year for BDE47, BDE209, and HBCDs, respectively (Figure 2). Total losses by sediment burial were the other dominant output pathway, with annual average losses of 45 ± 20, 83 ± 30, and 0.87 ± 0.66 kg/year for BDE47, BDE209, and HBCDs, respectively. Water-to-air transfer (12 ± 5.4 kg/year) was greater than water outflow for BDE47 (7.5 ± 3.1 kg/year). While for BDE209 and HBCDs, water-to-air transfer was negligible due to their low volatility. Our results indicate that water was not a permanent sink for the studied HFRs because a large proportion of the BDE47, BDE209, and HBCDs are transported to the sediment through sediment burial.

Due to the lack of concurrent measurements in lake water, sediment, tributaries, and WWTPs, direct comparisons are not possible for most of the study period. Therefore, we further extend the modeling using the method described in Figure S10 to predict the trends of BDE209 and HBCDs. The predicted concentrations can therefore be compared with measurements from the same year. For example, the estimated BDE209 concentrations in lake water in 2018 were 5.0 ± 2.4 pg/L, close to the measured value of 7.7 ± 1.1 pg/L in the samples taken from the same year (Table S7). Modeled BDE209 concentration in sediment in 2007 was 44 ± 80 ng/g dw, comparable with the values of 32–62 ng/g dw measured in the same year.^74^Figure S11 shows the time trends of input and output pathways, which suggests that the inter-media transfers for BDE209 have decreased over time. However, it is worth noting that there has been an increase in the multimedia transfer of HBCDs after 2012, which suggests potential risks to the ecosystem.

The inverse modeling was applied to a fugacity-based fish model^36^ to estimate the intake of food for lake trout and to investigate the mass balance of HFR concentrations in the lake water and fish. The results indicate that lake water was not the major input pathway for lake trout. For example, the influx of BDE47 from lake water was estimated at 8.08 × 10^–12^ mol/h, which was about four orders of magnitude lower than that from diet intake (2.39 × 10^–8^ mol/h). Although lake water was not the dominant input pathway for lake trout, the similar decreasing trends of PBDEs in the lake water (Table S7 and Figure S11) and lake trout suggest that the dietary intake could have decreased during the sampling period. This is reasonable as HFRs accumulated from water into the food chain and biomagnified through the food chain in Lake Ontario.^75^

**Figure 3:**
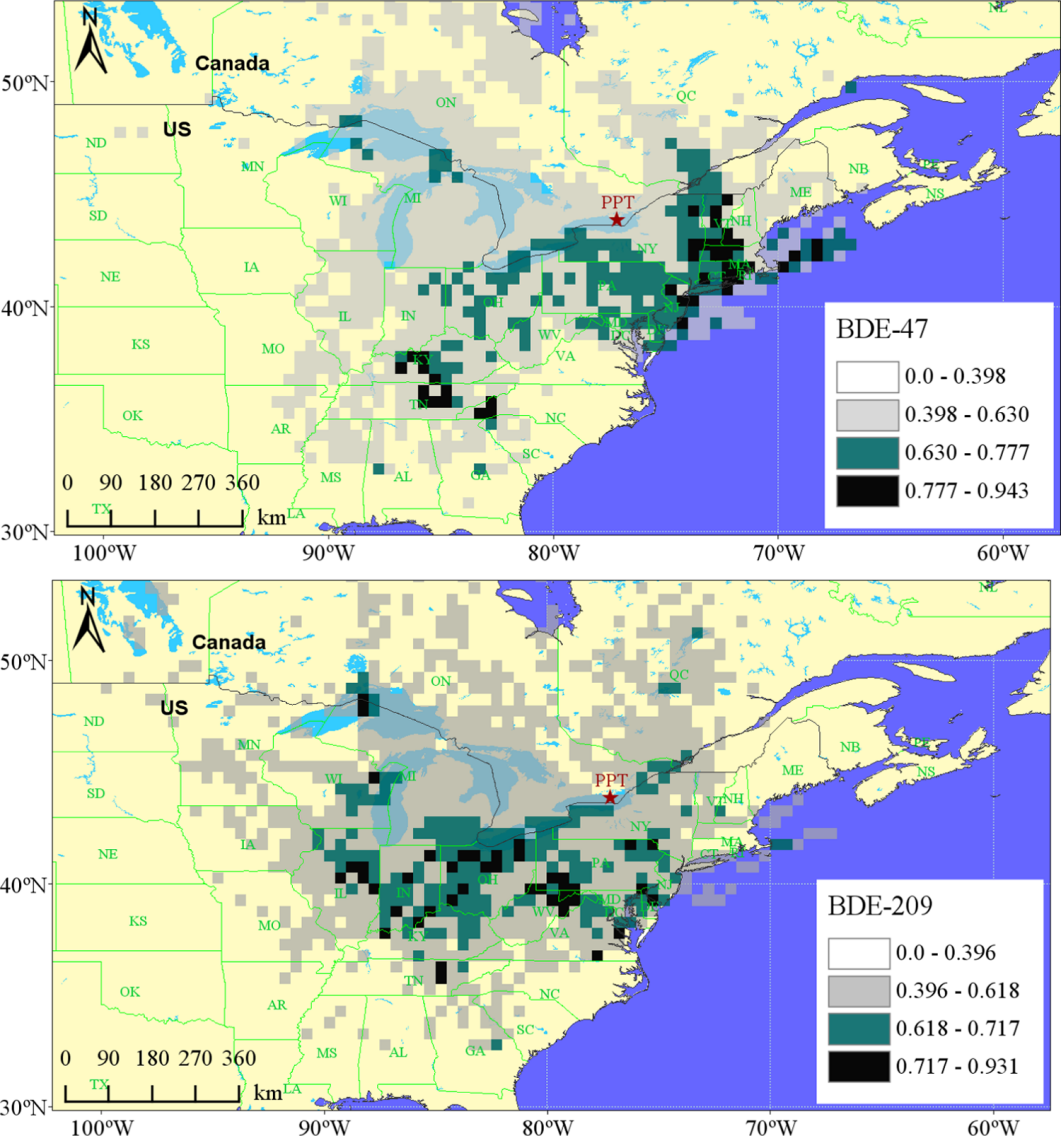
Weighted PSCF of BDE47 and BDE209 in the atmosphere at the PPT site. Regions with higher PSCF values, indicated by darker colors, are more likely to have emission sources.

The modified QWASI model results revealed that the atmosphere is an important source of lower-molecular-mass HFRs in Lake Ontario. Concentrations of BFRs in the atmosphere are also governed by air mass back trajectories. The source of atmospheric HFRs can be derived using the PSCF modeling, see the SI for details. The potential source regions of atmospheric BDE47 and BDE209 were identified to be mostly south of the sampling site from the US (Figure 3). Many studies have shown that concentrations of BFRs in the air are significantly correlated with the population and economies.^76,77^ While the US and Canada share deeply integrated economies, both population and gross domestic product in the US are about 8–10 times higher than that in Canada, making the US a stronger emission source of atmospheric HFRs than Canada to Lake Ontario.

## Limitations and Implications

There were some limitations of this study. First, only one regionally representative remote master station was included in the study; as the sampling site for air and precipitation is located downwind of the source, the atmospheric loadings to Lake Ontario could be underestimated. Total inputs to the lake were underestimated when the emissions from the other sources were not included in the model. Second, atmospheric deposition was not considered for BDE209 when performing PSCF. Nevertheless, a previous study found that most of the HFRs were detected in the finest aerosols with a size less than 0.95 μm, suggesting that HFRs were more likely to present in smaller particles compared to larger particles.^78^ The 1.0 μm particles containing HFRs can remain in the atmosphere for up to 300 days and can travel over thousands of miles.^79^ Third, there are uncertainties associated with the model; for example, the modeling results are sensitive to parameters such as chemical half-life, water volume, organic carbon content, sedimentation rate, and directly discharged into Lake Ontario (Figure S12). Accurately incorporating the values and accounting for the variability of these parameters in the modeling process is crucial for effectively simulating the fates of HFRs in lakes.

This study highlights the widespread occurrence of HFRs in Lake Ontario and their potential adverse effects on the ecosystem and human health. Our results indicate that all targeted HFRs were detected in air, precipitation, lake trout, and herring gull egg samples throughout the sampling periods. The widespread presence of HFRs in the lake may pose certain risks to the aquatic ecosystem if exposures increase further, as some of these chemicals can accumulate in the food chain and lead to reproductive and developmental abnormalities in fish and other aquatic organisms. Some HFRs also have the potential to harm human health, as they can be absorbed into the body through the inhalation of air, and the consumption of contaminated fish and water. We evaluated the temporal trends of PBDE congeners and AHFRs in these samples and observed general decreasing trends for PBDEs, while the trends for AHFRs were less clear. Our findings suggest that physical–chemical properties and emissions significantly influence the levels, profiles, and trends of HFRs.

As a first attempt at the multimedia analyses for emerging HFRs from the Great Lakes, we also identified the loadings from tributaries and wastewater effluent as the primary sources for the input pathway, while total transformations in the water and sediment are the dominant output pathway. These results are critical in assessing the environmental fate and behavior of HFRs, which have become ubiquitous in the environment. The trends analysis of HFRs in the atmosphere and biota from Lake Ontario can have significant implications for human health, the environment, regulations, and public awareness. Increasing levels of several alternative HFRs in the atmosphere could indicate a potential threat to human health and the aquatic organisms in the lake associated with exposure to these chemicals. This can lead to increased demand for safer and more sustainable products and put pressure on manufacturers to find safer alternatives to HFRs. The findings underscore the importance of continued monitoring of emerging HFRs in the Great Lakes and taking appropriate actions to reduce their potential risks if their concentrations continue to increase in the future.

## Supporting Information Available

Details of supplementary methods, results from principal component analysis (PCA), physical–chemical properties of target compounds, parameters used in the QWASI model, measured concentrations, predicted concentrations in sediment and water, calculated residence times, sampling sites, concentration profiles, temporal trends, correlation analyses, model workflow, trends of transportation flux, sensitivity analyses, and PCA plots (PDF)
