# Supplementary material for: Temporal Trends of Legacy and Current-Use Halogenated Flame Retardants in Lake Ontario in Relation to Atmospheric Loadings, Sources, and Environmental Fate
Source: Environ Sci Technol. 2023 Sep 11;57(38):14396–406. doi: 10.1021/acs.est.3c04876 (PMC10537453; doi:10.1021/acs.est.3c04876)
Supplement: Supplementary file 1 — es3c04876_si_001.pdf [file es3c04876_si_001.pdf]

Supporting Information for

Temporal Trends of Legacy and Current-Use Halogenated Flame Retardants in Lake Ontario in Relation to Atmospheric Loadings, Sources, and Environmental Fate

Wen-Long Li[1], Tana V McDaniel[2], Shane R de Solla[3], Lisa Bradley[2], Alice Dove[2], Daryl McGoldrick[2], Paul Helm[4], Hayley Hung[1,*]

[1] Air Quality Processes Research Section, Environment and Climate Change Canada, 4905 Dufferin Street, Toronto, Ontario M3H 5T4, Canada

[2] Water Quality Monitoring and Surveillance Division, Environment and Climate Change Canada, Burlington, Ontario, L7S 1A1, Canada

[3] Ecotoxicology and Wildlife Health Division, Environment and Climate Change Canada, Burlington, Ontario, L7S 1A1, Canada

[4] Ontario Ministry of the Environment, Environmental Monitoring and Reporting Branch, 125 Resources Road, Toronto, Ontario M9P 3V6, Canada

* Corresponding author:

Hayley Hung, phone: +1-416-739-5944, E-mail address: hayley.hung@ec.gc.ca

41 pages, 8 tables, 13 figures and 12 references.

Table of Contents

S1. Supplementary Methods S4

S1.1. Sampling S4

S1.2. Sample Analysis S7

S1.3. QA/AC S10

S1.4. HFRs in Tributaries and Wastewater Effluents S12

S1.5. Trend Analysis. S13

S1.7. PSCF Modelling S13

■ **S2. Supplementary results** S15

S2.1. Principal component analysis (PCA) results S15

S3. Supplementary Tables S16

Table S1. Physical-chemical properties of BDE47, BDE209, and HBCDs calculated by EPIWEB V4.1, Open (Quantitative) Structure-activity/property Relationship (OPERA) and provided from previous studies S16

Table S2. The mean and standard deviation (SD) of parameters used in one of the 1000 Monte Carlo Simulations to predict the concentrations of BDE47 in water and sediment S17

Table S3. Average, standard deviation (SD), and median concentrations and detection frequency (%det) of HFRs in air precipitation. S18

Table S4. Average, standard deviation (SD), and median concentrations (ng/g), and detection frequency (%det) of HFRs in lake trout and herring gull eggs. S19

Table S5. Summary of the total length, weight, age, lipid content (%Lipid), and moisture content (%Moisture) for lake trout. S20

Table S6. Summary of the percent lipid (%Lipid) and moisture content (%Moisture) for herring gull eggs. S21

Table S7. The predicted concentrations (mean ± standard deviation) in the lake water (pg/L) and sediment (ng/g dw). S22

Table S8. The calculated residence times (in days) for the water, sediment, and water-sediment system S23

S4. Supplementary Figures S24

Figure S1. Air and precipitation sampling sites (Point Petre), fish sampling sites (Cobourg, Port Credit, and Niagara on the Lake (NOTL)), and pooled egg sampling sites (Snake Island) from Lake Ontario. S24

Figure S2. Concentrations of HFRs in air, precipitation (Pricip), lake trout, and herring gull (HERG) egg samples collected from Lake Ontario S25

Figure S3. Trends of HFRs in air samples from Point Petre, Ontario, Canada. S26

Figure S4. Trends of natural logarithm concentrations of HFRs in precipitation from Point Petre, Ontario, Canada S28

Figure S5. Trends of natural logarithm concentrations of HFRs in lake trout from Lake Ontario. S30

Figure S6. Trends of natural logarithm concentrations of HFRs in herring eggs from Lake Ontario. S32
Figure S7. Correlations between HFR concentrations in the air and ambient temperature. S33
Figure S8. Correlation between the HFR concentrations in the precipitation and the ambient temperature. S34
Figure S9. Lake trout HFR concentrations vs fork length, weight, age, lipid, and moisture. S35
Figure S10. The workflow designed for implementing the modified QWASI involves estimating the temporal trends of HFR emissions and concentrations in water and sediment. S36
Figure S11. Trends of Transportation flux for (a, b) BDE209, and (c, d)HBCDs in Lake Ontario from 2004-2019. .... S37
Figure S12. Sensitivity analyses on the parameters affecting the predicted concentrations of BDE47 in water (pg/L) and sediment (n/g dw). S38
Figure S13. Principal component analysis (PCA) on selected HFRs in the air, precipitation (Pric), fish, and egg samples. S39
S5. References: S40

S1. Supplementary Methods

S1.1. Sampling

Details on the sampling of air and precipitation were also summarized in previous studies.[1-3] Brief descriptions are given below.

(1) Siting criteria

All Master Stations are background sites close to the shore of the lakes and are considered 'representative' of the area. 'Representative' means that these sites are situated so that they are not biased by the impacts of one or just a few nearby sources. All variance from these criteria should be noted on a standard siting checklist and a site map including a 1 km radius around the site. In general, only minor deviations from these criteria should be allowed for Master Stations. More significant deviations are permitted for satellite sites; however, there should be no more than one major source nearby and it should be downwind during prevailing winds.

Sites should be at least 40 km from major sources such as larger urban centers (population>10,000), heavy industry, chemical plants, foundries, steel mills, smelters, refineries, pulp, and paper mills) or other major sources of airborne metals or organic chemicals (large incinerators, power plants emitting more than 10,000 tons/year SO2, NOX or 100 kg/year total PAH, major airports, large sewage treatment plants).

Sites should be at least 10 km from other important sources such as urban areas (population = 1,000 to 10,000), mining and manufacturing facilities, major highways, commercial areas, electrical transfer stations, or smaller sources (lower by a factor of 10).

Sites should be at least 1 km from local sources such as vehicles or boat traffic (>30 vehicles/hr), farms and tilled fields (in regions where farming is not predominant land use),

fuel or chemical storage areas, landfills, sewage lagoons or small towns (pop.<1,000). The use of pesticides in farming operations within 1 km of the sites should be documented.

Sites should be at least 250 m from single residences, parking lots, grazing animals, public roads (<30 vehicles/hour), and other sources in the immediate area of the samplers. Single residences within 1 km of the site should be downwind during prevailing winds or outside of a 22.5° sector on each side of the prevailing wind direction. For all residences within 500 m of the site, the type of heating and fuel usage, open burning, and usage of household chemicals should be documented.

Samplers should be sited on open terrain (slope <15%) with regionally representative ground cover (preferable grass with a height of no more than 20 cm). The samplers should subtend a vertical angle of less than 30° (2:1 fetch to obstruction height) with any obstruction (trees, towers, power lines). This should be increased to 10:1 for solid structures. In areas with high snowfall, the site should be sheltered by trees at a distance between 2.5 and 4 times their height. The samplers should be on 1 m platforms or no higher than the maximum snowpack.

Sites should be no more than 1 km from the lakeshore. Where there is a choice, sites should be situated so that prevailing winds are onshore from the lake. Protection by trees from wind and lake spray at a distance from 2.5 to 4 times tree height is also desirable.

Sites should have all-weather access, electrical power (200 amp, 125 v), and a security fence if not otherwise secured. Sites should be large enough to allow a 2 m spacing between samplers.

No development (industry, construction) should be planned in the vicinity of the site (1 km) and the site owner should agree to continuous operation of the site for at least 5 years.

(2) Air and Precipitation Sampling

The General Metal Works PS-1 high-volume sampler was used to collect air samples, with a sampling air volume of approximately 350 m^3 over 24 hours and a flow rate of 0.24 m^3/min. The gas-phase sample was taken using a pre-cleaned polyurethane foam (PUF, Pacwill Environmental) plug. The particle-phase sample was taken using a prebaked glass fiber filter (GFF, 10.2 cm diameter, Gelman A/E Microfiber). At Point Petre on Lake Ontario, a total of 263 pairs of gas- and particle-phase samples were collected. Air samples were taken once every 12 days from January 2005 to December 2012 and changed to once every 36 days until now.

Precipitation samples were taken monthly using a MIC-B sampler (Meteorological Instruments of Canada, Thornhill, ON). From April 2006 to August 2018, a total of 157 precipitation samples were collected at the Point Petre site. Dichloromethane (250 mL) was added to the sample as a sampling medium to preserve and extract the sample at the time of sampling. After monthly collection, the high-density polyethylene (HDPE, 2.0 L) bottles were used to store the sample during shipment. Sample volume greater than 2.0 L was measured and discarded from further analysis. All samples were stored in the lab at −10 °C until sample analysis.

(3) Lake Trout Sampling

Adult lake trout were sampled in late summer from Canadian sites on Lake Ontario by gillnetting at depths from 20 to 60 meters. Fish were placed in contaminant-free food-grade

plastic bags, frozen at -20C, and transported back to the laboratory. Morphometric measurements such as weight, length, and sex were noted and fish were aged based on tags and/or otoliths. Only fish in the four to six-year age range were included in this analysis as PBDEs may continue to accumulate with age. Lake trout (Salvelinus namaycush) were collected from 3 sites in the western and central basin of Lake Ontario (refer to SI for locations) between 1997 and 2018 for the analysis of HFRs. A total of 243 fish samples were analyzed as part of the ECCCs Fish Contaminant Monitoring and Surveillance Program (FCMSP) to support the Chemicals Management Plan (CMP), which monitors contaminants in the Great Lakes and across Canada.[4]

(4) Herring Gull Egg Sampling

The Great Lakes Herring Gull Monitoring Program has been collecting Herring Gull eggs annually since 1975 from 15 colonies throughout the Great Lakes and connecting channels, and this study utilized data from Snake Island (44.1908, -76.5432) on Lake Ontario. Thirteen to fifteen freshly incubated eggs were generally collected in late April to early May each year. Eggs were collected soon after the last egg was laid and clutches with two or fewer eggs were generally not targeted for egg collection. Eggs were haphazardly collected from each clutch. After collection, egg contents were placed in acetone- and hexane-rinsed amber jars and frozen prior to chemical analysis.

S1.2. Sample Analysis

(1) Air Sample Analysis

PUF and GFF sampling media were analyzed separately for HFR Composition. The PUFs and GFFs were extracted by the Soxhlet and a Dionex ASE200 system, respectively.[1] The extracts of PUFs (hexane) and GFFs (hexane and acetone mixture (7: 3; v/v)) were

passed through anhydrous sodium sulfate and silica column for clean-up. Levels of BDE209 and decabromodiphenyl ethane (DBDPE) were analyzed by gas chromatography-electron capture detection (GC-ECD) using a short Rtx-1614 column (15 m × 0.25 mm i.d. × 0.10 μm film thickness) because of the high sensitivity. Analysis of other HFRs in the air samples was conducted by GC- mass spectrometry in electron capture negative ion mode, using a DB-5 column (30 m × 0.25 mm i.d. × 0.25 μm film thickness) for the other HFRs.

(2) Precipitation Sample Analysis

First, the aqueous phase was separated from the dichloromethane solvent in a separatory funnel, and the precipitation volume was measured. Next, the aqueous phase was extracted twice using fresh dichloromethane. The combined extracts were then concentrated to 3.0 mL by rotary evaporation, and the solvent was exchanged for isooctane. Afterward, the extract was purified on 3.5% (w/w) water-deactivated silica gel and was first eluted using hexane followed by a 1:1 mixture of hexane and dichloromethane. The extracts were then concentrated to 1.0 mL using nitrogen blowdown. Finally, the extracts were analyzed for HFRs using dual-capillary gas chromatography with electron capture detection on an Agilent 6890 gas chromatograph.

(3) Lake trout Sample Analysis

Whole fish, including gut contents, were homogenized using a HOBART commercial meat grinder, and sub-sets samples were stored at -20°C or -80°C until analysis. For PBDEs, and other brominated flame retardants, samples from all years were analyzed by commercial laboratory SGS/AXYS (Sidney, British Columbia, Canada) using method MLA-033 equivalent to EPA Method 1614A as described in a previous study.[5] In brief,

samples were extracted, cleaned by column chromatography, analyzed by high-resolution gas chromatography with high-resolution mass spectrometric detection (HRGC/HRMS), and quantified using isotope dilution. HBCDD was analyzed using method MLA-070 whereby samples were soxhlet extracted with DCM, cleaned in a gel permeation column (GPC) followed by Florisil column, and analyzed using high-performance liquid chromatography (LC-MS/MS) on a reversed-phase C18 column coupled to a triple quadrupole mass spectrometer in MRM mode. The remaining flame retardants were analyzed by method MLA-106 with involved soxhlet extraction, cleaned up by GPC, and analyzed by GC-(electron capture negative ion (ECNI)-MS. Quantification was performed by internal standard/ isotope dilution.

In addition, a subset of samples in 2013 was analyzed by the National Laboratory for Environmental Testing (NLET) for brominated flame retardants using similar methods, ie soxhlet extraction, lipid removal by gel permeation chromatography, silica gel clean up and analyzed using HRGC-(ECNI)-MS; with quantification was based on multi-point calibration standard solutions.

(4) Herring Gull Egg Sample Analysis

Sample extraction and instrumental analysis of BFRs in gull eggs are described in detail in a previous study.[6] Approximately 2.0 g of the homogenated egg (wet weight, ww) was spiked with BFR internal standards. Lipid content was measured using gravimetric determination from 10% of the extract, the remaining of which was subjected to gel-permeation chromatography, followed by cleanup on an SPE silica gel (SiOH) cartridge. The sample was eluted with 8 mL of 5% DCM/HEX (v/v) from the SPE column. The final extract volume was adjusted to approximately 250 μL for instrumental analysis. BFRs were

analyzed using an Agilent 6890 gas chromatography (Agilent Tech., Palo Alto, CA, USA) coupled to a single quadrupole mass analyzer (Agilent 5973 MS) in the electron capture negative ionization (ECNI) mode. The column used was a 15-m DB-5 HT column (0.25 mm i.d., 0.1 µm, J&W Scientific, Agilent Tech.). Brominated FR quantification was achieved via selected ion monitoring (SIM) for $^{79}Br^-$ and $^{81}Br^-$, except for BDE-209 (*m/z* 487) and $^{13}C_{12}$-BDE-209 (*m/z* 495). From 2014 onwards, the analyses were identical except that extracts were analyzed using single quadrupole GC-MS (Agilent 7890B/5977A). Results were recovery corrected and also corrected for background contamination by subtracting the concentration values of the associated method blank.

S1.3. QA/AC

(1) *QA/AC for Air and Precipitation samples*

Six field blanks were collected each year at every site by placing a sampler head with a clean sampling media in a high-volume air sampler and immediately removing it without drawing air through the sampler. Laboratory and field blanks were processed in the same way as the samples to determine the contamination introduced during extraction and clean-up; and by handling, shipping, and storage, respectively. Blank clean samples were spiked with HFR standards and processed as air samples. A silica column was spiked with the same HFR standards and taken through the clean-up procedure for each sample batch. The recoveries of HFRs ranged from 75% to 97% for PBDEs and from 60% to 99% for AHFRs.

Research Data Management and Quality Control SystemTM (RDMQTM) is an interactive SAS-based system that evaluates and flags data. The RDMQTM evaluates raw data by assigning flags based on sample quality (duration and volume of sample, meteorological conditions, presence of unusual anthropological or natural conditions at the

site, and adherence to field and laboratory protocols). Data are flagged and may be invalidated if they are of low sample quality. Statistical analysis of field blank concentrations was used to assess background method concentrations and to determine method detection limits. Statistical analysis of samples was used to evaluate the percentage of quantifiable samples and to identify and investigate outliers. The data were also manually reviewed and identified for any anomalies present by plotting time series of ambient concentrations and quality indicators such as blank levels and method detection limits. Finally, summaries of quality control activities such as the analysis of common reference standards and matrix spikes were used to provide information about the quality of the final dataset. The summarized data were approved by the data management team and the principal investigator. The raw data have undergone several evaluation steps before being accepted as valid data.

(2) QA/AC for Lake trout and herring gull egg samples

All samples are spiked with isotope-labeled surrogate standards to provide information on the efficiency of measurement and recovery during analysis and in some cases (samples analyzed by SGS-AXYS) for quantification of individual parameters by isotope dilution. Surrogate standards were also used to track extraction efficiency and clean-up of samples prior to analysis. Solvent blanks were included with every batch of samples, generally with a ratio of one blank to every 20 samples along with duplicate sample measurements. Reference standards and matrix spikes were also used to inform the quality of the analysis and final data set. QC acceptance criteria were set for calibration, spiked matrix recovery, and blanks for the dataset to be excepted. Data was not blank subtracted.

Method blanks for herring gull eggs were processed to monitor interferences and contamination. An in-house Reference Material consisting of homogenated double-crested cormorant eggs was analyzed for HFRs with each batch of samples. Replicate analyses (n = 4) were made on standard additions to the SRM to evaluate the analytical precision and accuracy for non-PBDE FRs. Average recovery efficiencies of BFR standards were generally > 95%. Reported analyte concentrations were corrected based on the recovery of internal standards, and presented as ng/g ww. For each FR, the method limit of quantification (MLOQ), defined as a minimum amount of analyte producing a peak with a signal-to-noise ratio (S/N) of 10, and the method limit of detection (MLOD), defined as S/N = 3, are listed in **Table S1**.

S1.4. HFRs in Tributaries and Wastewater Effluents

Details on the analyses of HFRs in tributaries and wastewater effluents can be found in a previous study.[7] PBDEs were analyzed from the sampling campaigns of the Toronto Tributary Assessment (TTA) project. A total of 6 streams (Etobicoke Creek, Mimico Creek, Humber River, Don River, Highland Creek, and Rouge River) that discharge into Lake Ontario were included in calculating the tributary loadings. Annual loadings from tributaries were estimated from water HFR concentrations (ng/L) and the water discharge rate (m^3/d). Final wastewater effluent samples were collected by Ontario Ministry of the Environment and City of Toronto staff at three City of Toronto WWTPs that discharge directly to Lake Ontario. Three major WWTPs (Highland Creek, Ashbridges Bay, and Humber Bay) were selected to calculate the loadings using the 24-h composite effluent HFR concentrations (ng/L) and daily discharge rate (m^3/d). Stream and wastewater samples were analyzed at the Laboratory Services Branch, Ontario Ministry of the Environment in

Toronto, Ontario for a suite of metals and conventional water quality parameters and three classes of persistent organic pollutants. The PBDEs were also analyzed by GC-HRMS combined with internal standard and isotope dilution for quantification as outlined in method E3430. Due to the difficulties encountered with trace-level analyses, meticulous quality assurance and control measures were implemented during the sampling and analysis of organic compounds. Travel blanks were taken into the field during sample retrievals at a frequency of one per sampling run, and upon retrieval, they were stored and treated as samples. Each set also underwent a laboratory process. To compensate for losses during the extraction process and instrumental analysis variations, mass-labeled surrogate standards were introduced into the resin cell of each sample before processing.

S1.5. Trend Analysis.

The long-term trends for HFRs in air and precipitation were analyzed by the Digital Filtration (DF) method. Linear regression of ln transformed data was used for trend analysis in herring gulls and lake trout. In the DF method, the Fourier components and a smoothing Reinsch-type cubic spline were used to fit the seasonal cycle and temporal trends of the HFR concentrations, respectively. Outlier data that have concentrations greater than 3 times the standard errors away from the fitting curve were removed from the DF analysis. The short-term and long-term variations were analyzed by the two Butterworth digital filters using the short-term and long-term cutoff periods. The apparent first-order halving time ($t_{1/2}$) or doubling time (t_2) was derived by dividing ln 2 with the regression slope of sampling years and the concentrations of HFRs in the samples.

S1.7. PSCF Modelling

The potential source contribution function (PSCF) modeling, which is based on the backward trajectories, was applied to derive the potential upwind source regions. The air

back-trajectories were calculated using the Hybrid Single-Particle Lagrangian Integrated Trajectory (HYSPLIT) model, which is available on the National Oceanic and Atmospheric Administration (NOAA) website. For each sample, a total of 18 backward trajectories were generated, which consisted of 6 starting times during each sampling event (from 9 am on the first day until 5 am on the second day with a 4-hour interval) and 3 starting altitudes (10, 100, and 500 m above the ground level) for each starting time. The NCEP/NCAR Reanalysis data archives were used to generate the five-day backward trajectories.

The geographical region of longitude 50-130 °W, and latitude 20-70 °N was selected to cover most of the trajectories and was divided into small grid cells of 0.5° longitude × 0.5° latitude. PSCF calculations were performed for each grid cell by,

$$PSCF_{ij} = m_{ij}/n_{ij} \qquad \text{(SI-4)}$$

where n_{ij} represents the locating in the ij^{th} grid cell, and m_{ij} refers to endpoint numbers of trajectories that are associated with concentrations greater than arbitrary preset criteria, which is the median concentration of PAHs for each source sector in this study. Concentrations of PAHs from each source sector were assigned to each trajectory.

The arbitrary weight function (W_{ij}), which is necessary to minimize the high uncertainties caused by the small number of trajectory endpoints in some grid cells, is applied to down-weight the PSCF,

$$W_{ij} = \begin{cases} 0.20 & if \quad n_{ij} < n_{ave} \\ 0.42 & if \quad 1.5n_{ave} > n_{ij} > n_{ave} \\ 0.70 & if \quad 3n_{ave} > n_{ij} > 1.5n_{ave} \\ 1.00 & if \quad n_{ij} > 3n_{ave} \end{cases} \qquad \text{(SI-5)}$$

where n_{ave} is the average number of endpoints in the grid cells.

■ S2. Supplementary results

S2.1. Principal component analysis (PCA) results

We selected 11 HFRs based on their relative abundance and had high detection frequency in all four environmental media. As shown in **Figure S13(a)**, a total of 2 principal components (PC) which explained a significant portion of the variance were obtained for the samples. Most of the target compounds (BDE209, 99, 85, 183) showed high loadings in the first principal component (PC1, explained 66% of the total variance), indicative of a similar pattern of these compounds in the samples. BDE47, 99, 100, 49, 66, and HBCDs showed higher loadings in PC2, suggesting that their distribution patterns are different from other HFRs.

Samples with similar BFR compositional patterns will cluster together. We can identify key samples and relationships amongst groups of samples by the distribution of samples in the score plot (**Figure S13b)**. PC1, which explained 66% of the variation, differentiated herring gull eggs from the other three environmental media, whereas PC2, which explained 14% of the variation, differentiated the biotic media from the abiotic media.

S3. Supplementary Tables

Table S1. Physical-chemical properties of BDE47, BDE209, and HBCDs calculated by EPIWEB V4.1, Open (Quantitative) Structure-activity/property Relationship (OPERA) and provided from previous studies.

	BDE47	BDE209	HBCD
CAS	5436-43-1	1163-19-5	3194-55-6
Molecular Weight	485.8	959.17	641.7
Log *K*ow	6.80 [a]	9.97 [a]	6.06 [a]
Boiling Point (deg C)	406 [b]	590 [b]	462 [b]
Melting Point (deg C)	162 [b]	255 [b]	180 [b]
Vapor Pressure (Pa)	2.0E-04 [a]	8.9E-8 [a]	2.5E-05 [a]
Subcooled liquid Vapor Pressure (mm Hg)	1.6E-06 [b]	4.7E-09 [b]	1.6E-05 [b]
Subcooled liquid Vapor Pressure (Pa, 25 deg)	2.1E-04 [b]	6.3E-07 [b]	2.1E-03 [b]
Water Solubility at 25 deg C (mg/L)	7.2E-02 [a]	2.2E-03 [a]	1.2E-01 [a]
Henrys Law Constant (atm-m^3/mole)	8.5E-06 [b]	4.5E-08 [b]	6.5E-06 [b]
Henrys Law Constant (Pa-m^3/mole)	1.1E+01 [b]	4.5E-03 [b]	4.7E+00 [b]
Log K_{oa} (experimental database)	10.1 [a]	14.8 [a]	10.3 [a]
Log *K*aw	-3.3 [a]	-4.8 [a]	-4.3 [a]
K_p (particle/gas partition coef. (m^3/ug))	0.0142 [b]	4.75 [b]	0.00141 [b]
Fraction sorbed to airborne particulates	0.533 [b]	0.997 [b]	0.101 [b]
Overall OH Rate Constant (cm^3/molecule-sec)	1.0 E-12 [b]	0.034 E-12 [b]	5.0 E-12 [b]
Atmospheric Oxidation Half-Life (days)	11 [b]	318 [b]	2.1 [b]
Log *K*oc (L/kg (MCI method))	4.12 [b]	5.44 [b]	4.99 [b]
Log BCF from regression-based method (L/kg wet-wt)	4.13 [b]	1.62 [b]	3.76 [b]
Log Biotransformation Half-life (HL, days)	1.56 [b]	2.77 [b]	1.49 [b]
Biotransformation Half-life (HL, hr)	NA	12309 [c]	6460 [c]
Log BAF Arnot-Gobas method (upper trophic BAF)	5.83 [b]	0.84 [b]	5.59 [b]
Half-Life from Model River (hr) Volatilization	154 [b]	40750 [b]	34.8 [b]
Half-Life from Model Lake (hr) Volatilization	1869 [b]	444800 [b]	592 [b]
Level III Fugacity Model Half-Life air (hr)	256 [b]	7620 [b]	51 [b]
Level III Fugacity Model Half-Life water (hr)	4320 [b]	4320 [b]	1440 [b]
Level III Fugacity Model Half-Life soil (hr)	8640 [b]	8640 [b]	2880 [b]
Level III Fugacity Model Half-Life sediment (hr)	38900 [b]	38900 [b]	13000 [b]
Persistence Time (hr)	5740 [b]	7260 [b]	2570 [b]

[a] Reference: Li et al., 2022 [8]
[b] Reference: EPIWEB V4.1.
[c] Reference: Open (Quantitative) Structure-activity/property Relationship (OPERA) V2.9.

Table S2. The mean and standard deviation (SD) of parameters used in one of the 1000 Monte Carlo Simulations to predict the concentrations of BDE47 in water and sediment.

Notes	Unit	Mean	SD
Henry's Law Constant	Pa.m^3/mol	10.7	2.2
Half-Lives in Water	hours	4400	890
Half-Lives in Sediment	hours	40000	8100
*K*ow	NA	6.3E+06	1.3E+06
Air-Water (*K*aw)	NA	5.5E-04	1.1E-04
Subcooled Liquid Vapor Pressure	Pa	2.1E-04	4.1E-05
Water Surface Area	m^2	1.9E+10	3.8E+09
Water Volume	m^3	1.6E+12	3.3E+11
Sediment Active Layer Depth	m	4.9E-03	1.0E-03
Aerosols in Air	ug/m^3	30	6.1
Solids in Water Column	mg/L	0.64	0.13
Solids in Inflow Water	mg/L	24	4.6
Solids in Precipitation	mg/L	9.5	1.9
Solids in Sediment	vol/vol	0.15	0.03
Density-Aerosol	kg/m^3	1500	300
Density-Particles in Water	kg/m^3	2400	480
Density-Particles in Precipitation	kg/m^3	2400	490
Density-Sediment Solids	kg/m^3	2400	470
OC in Particles in Water (lake)	mass/mass	33%	6.4%
OC in Particles in Water (inflow)	mass/mass	33%	6.7%
OC in Particles in Precipitation	mass/mass	4.1%	0.78%
OC in Sediment Solids	mass/mass	4.1%	0.78%
OC in Resuspended Sediment	mass/mass	3.5%	0.71%
Water Inflow	m^3/h	2.2E+07	4.4E+06
Water Outflow	m^3/h	2.4E+07	4.9E+06
Sedimentation	g/m^2.day	1.40	0.28
Sediment Burial	g/m^2.day	0.60	0.12
Sediment Resuspension	g/m^2.day	0.61	0.13
Aerosol Deposition	m/h	7.2	1.4
Scavenging Ratio	vol air/vol rain	2.0E+05	3.9E+04
Rain Rate	m/y	1.1	0.21
MTC Volatilization air side	m/h	1.0	0.20
MTC Volatilization waterside	m/h	1.0E-02	2.0E-03
MTC Sediment-water Diffusion	m/h	4.0E-04	8.1E-05

Table S3. Average, standard deviation (SD), and median concentrations and detection frequency (%det) of HFRs in air precipitation.

	Air	pg/m^3			Precip	ng/L		
	Mean	SD	Med	%det	Mean	SD	Med	%det
BDE17	0.17	0.14	0.14	50	0.0088	0.0028	0.0075	6.4
BDE28	0.36	0.33	0.26	75	0.016	0.008	0.013	11
BDE47	3.7	4.8	2.1	99	0.075	0.16	0.049	97
BDE49	0.22	0.19	0.15	67	0.0085	0.011	0.0053	41
BDE66	0.14	0.15	0.089	39	0.018	0.0078	0.019	9.0
BDE71	0.17	0.14	0.088	5.7				
BDE85	0.20	0.47	0.065	37	0.013	0.018	0.0082	42
BDE99	2.1	5.3	1.1	99	0.11	0.20	0.081	96
BDE100	0.54	1.1	0.29	94	0.029	0.054	0.018	72
BDE138	0.18	0.24	0.058	6.8	0.029	0.013	0.026	14
BDE153	0.28	0.76	0.091	47	0.025	0.026	0.019	53
BDE154	0.24	0.67	0.082	53	0.022	0.033	0.017	57
BDE183	0.21	0.35	0.090	18	0.024	0.028	0.017	76
BDE190	0.98	1.1	0.98	0.80	0.026	0.017	0.024	12
BDE209	2.3	6.5	0.97	89	0.61	1.5	0.31	92
BTBPE	0.16	0.19	0.070	9.1	0.41	0.28	0.30	5.8
DBDPE	1.3	2.3	0.35	12	1.3	2.3	0.50	17
DPTE	0.16	0.19	0.080	18				
EHTBB	1.5	2.8	0.47	18				
HBB	0.22	0.24	0.16	32				
HBCDs	2.1	4.6	0.38	5.3	1.9	4.7	0.37	62
PBEB	0.049	0.033	0.039	17	0.0013	0.0011	0.0009	13
PBT	0.12	0.23	0.057	34				
ATE	0.55	1.1	0.22	31				
BATE	0.06	0.03	0.048	11				
synDP	2.4	8.8	0.31	53				
antiDP	5.9	22	0.72	59				

Note: HBCDs represent the sum of three isomers.

Table S4. Average, standard deviation (SD), and median concentrations (ng/g), and detection frequency (%det) of HFRs in lake trout and herring gull eggs.

	Trout	ng/g			Egg	ng/g		
	Mean	SD	Median	%det	Mean	SD	Median	%det
BDE17					0.97	1.3	0.43	75
BDE28					1.5	0.87	1.3	100
BDE47	40	31	30	100	83	27	85	100
BDE49	2.2	1.2	1.9	100	0.73	0.32	0.73	92
BDE66	1.0	0.68	0.83	100	1.4	0.82	1.0	100
BDE71	0.10	0.10	0.073	100				
BDE85	0.01	0.01	0.01	99	1.2	1.2	1.0	83
BDE99	11	6.8	9.6	99	82	29	73	100
BDE100	9.8	5.4	8.8	100	41	11	41	100
BDE138					1.0	0.55	1.0	83
BDE153	3.5	2.0	3.0	100	36	14	35	100
BDE154	5.2	2.6	4.6	100	22	7.2	21	100
BDE183	0.09	0.050	0.07	100	2.3	0.77	2.2	92
BDE190	0.0033	0.0030	0.0025	100				
BDE209	0.26	0.45	0.14	100	9.3	9.1	5.9	92
BTBPE					0.49		0.49	8.3
DBDPE	0.49	1.2	0.08	65				
DPTE	3.1	1.1	3.0	34				
EHTBB	0.23	0.32	0.060	34				
HBB	0.010	0.086	0.0010	75	0.32	0.090	0.29	25
HBCDs	4.7	2.8	4.0	82	12	16	5.8	92
PBEB	0.0006	0.001	0.0002	66				
PBT	0.014	0.021	0.003	34				
ATE	0.020	0.024	0.011	34				
BATE	0.055	0.090	0.004	34				
synDP	0.038	0.046	0.012	34	0.29	0.32	0.20	75
antiDP	0.57	0.31	0.53	34	1.3	2.0	0.53	100

Note: HBCDs represent the sum of three isomers.

Table S5. Summary of the total length, weight, age, lipid content (%Lipid), and moisture content (%Moisture) for lake trout.

Year	Total Length (cm)	Total weight (kg)	Age	%Lipid	%Moisture
1997	62	2.4	5.0	21	--
1998	57	1.9	4.0	18	--
1999	62	2.5	5.0	19	--
2000	67	3.0	6.0	17	--
2001	58	2.1	4.5	18	--
2002	61	3.1	5.0	18	--
2003	59	2.4	5.0	21	--
2004	55	1.8	5.0	13	--
2005	60	2.7	5.0	19	--
2006	60	2.4	5.0	17	--
2007	59	2.4	4.5	15	--
2008	61	2.6	5.0	16	--
2009	58	2.6	4.0	18	66
2010	60	3.3	5.0	17	64
2011	54	1.7	4.0	15	70
2012	65	3.1	5.0	18	63
2013	61	2.8	5.0	18	65
2014	65	3.3	5.0	17	64
2015	62	2.3	5.5	15	67
2017	61	2.6	5.0	19	66
2018	66	3.3	6.0	--	--
2019	59	2.5	6.0	21	62

Table S6. Summary of the percent lipid (%Lipid) and moisture content (%Moisture) for herring gull eggs.

Year	%Lipid	%Moisture
2008	6.2	80
2009	8.0	77
2010	7.3	77
2011	7.7	79
2012	8.3	79
2013	8.0	76
2015	8.7	77
2014	7.8	78
2016	8.9	75
2017	8.7	76
2017	7.2	78
2018	8.1	77

Table S7. The predicted concentrations (mean ± standard deviation) in the lake water (pg/L) and sediment (ng/g dw).

Year	Water	Sediment	Water	Sediment	Water	Sediment	Water	Sediment
	BDE209				HBCD			
	QWASI	QWASI	Reference	Reference	QWASI	QWASI	Reference	Reference
2007	37±18	43±77		32-62 [c]	17±4.5	0.88±1.7		0.84-3.1 [c]
2010	20±9.2	23±40		12±10 [a]	6.5±2.2	0.30±0.09		
2011	17±8.5	20±38	17±11 [d]		4.2±1.4	0.19±0.04	4.2±0.2 [d]	
2018	4.8±2.4	5.6±10	7.7±1.1 [b]		20±6.2	0.92±0.28	<30 [b]	

[a, b] Reference: Unpublished data. Lake water and sediment concentrations were both provided by the Water Quality and Monitoring Surveillance Division of Environment and Climate Change Canada. Sampling and analytical methods can be found in previous studies.[6, 9]
[c] Reference: Yang et. al., 2012 [10]
[d] Reference: Venier et. al., 2014[9]
Note: It is important to highlight that the halving time or doubling times of BDE47, BDE209, and HBCD in precipitation were utilized to estimate their concentrations in the absence of direct measurements for a given year. Additionally, due to the absence of concurrent measurement in lake water, sediment, tributaries, and WWTPs, extrapolation techniques based on these halving or doubling times were used to calculate the discharges from the tributaries and wastewater treatment plants (WWTPs) for a given year without concurrent measurement. Such methods could be used to approximate concentrations when direct measurements are unavailable. Despite the limitations, our study provides valuable insights into the behavior and fate of HFRs in the study area and can contribute to future monitoring and management endeavors.

Table S8. The calculated residence times (in days) for the water, sediment, and water-sediment system.

System		BDE47	BDE209	HBCD
Water system	Average	237	243	86
	SD	0.52	0.55	0.16
Sediment system	Average	1331	1331	631
	SD	1.46	1.46	0.98
Water-Sediment system	Average	859	1131	148
	SD	1.53	1.59	0.34

S4. Supplementary Figures

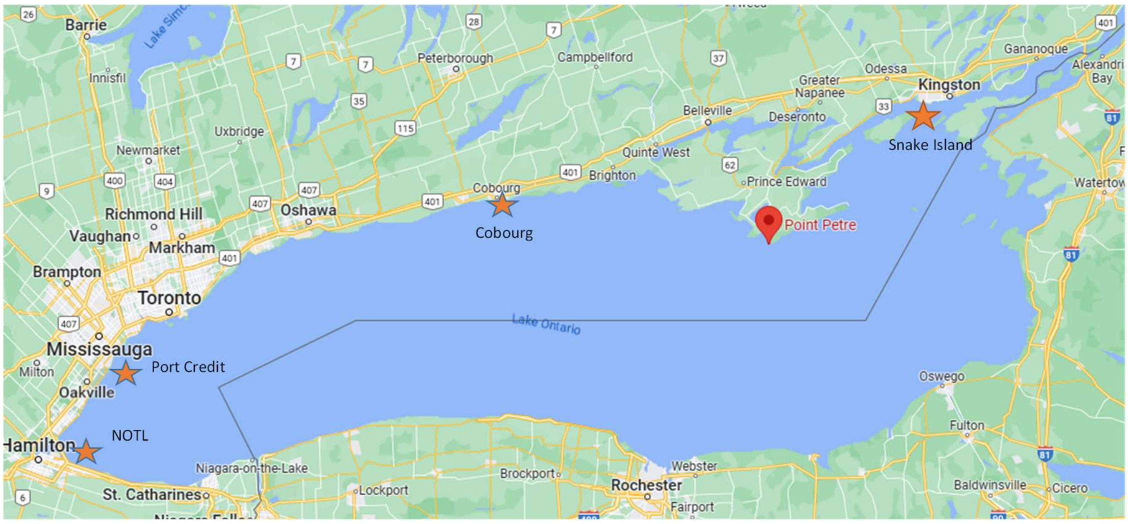

Figure S1. Air and precipitation sampling sites (Point Petre), fish sampling sites (Cobourg, Port Credit, and Niagara on the Lake (NOTL)), and pooled egg sampling sites (Snake Island) from Lake Ontario.

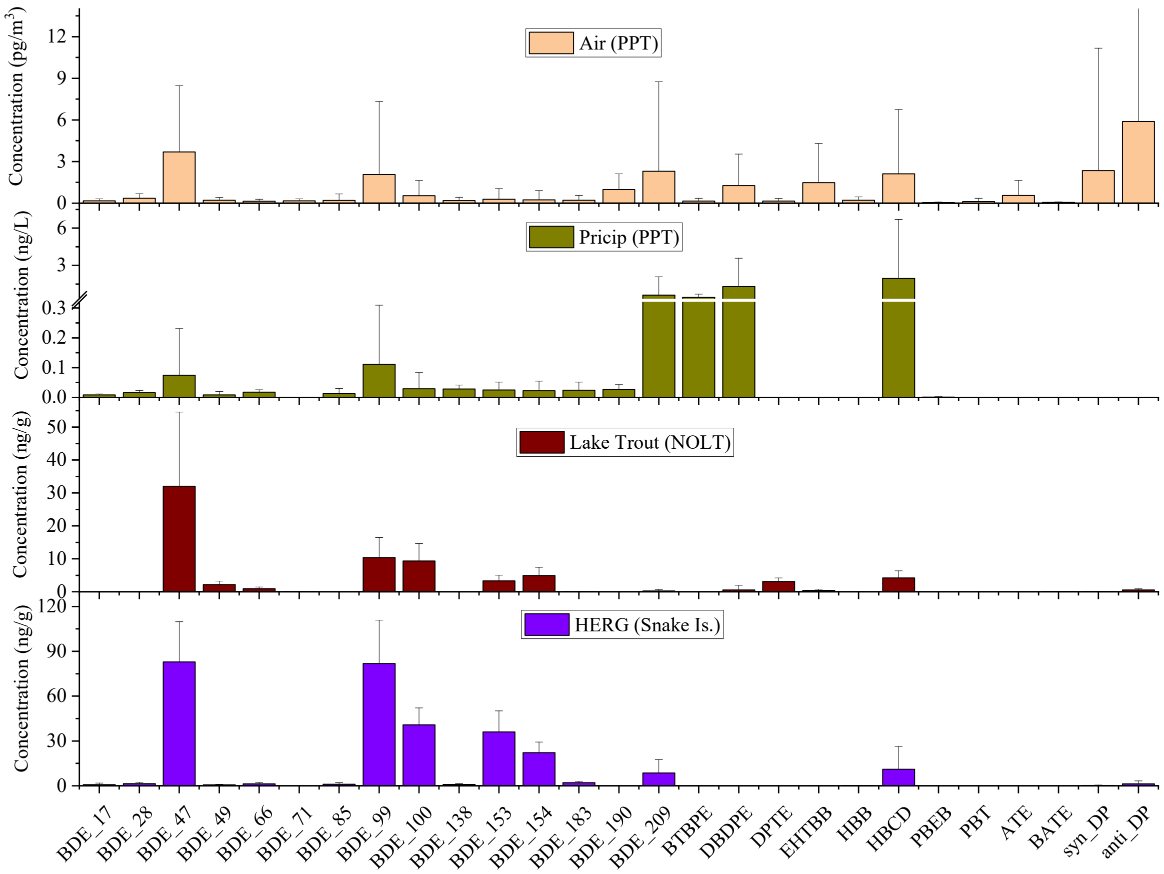

Figure S2. Concentrations of HFRs in air, precipitation (Pricip), lake trout, and herring gull (HERG) egg samples collected from Lake Ontario.

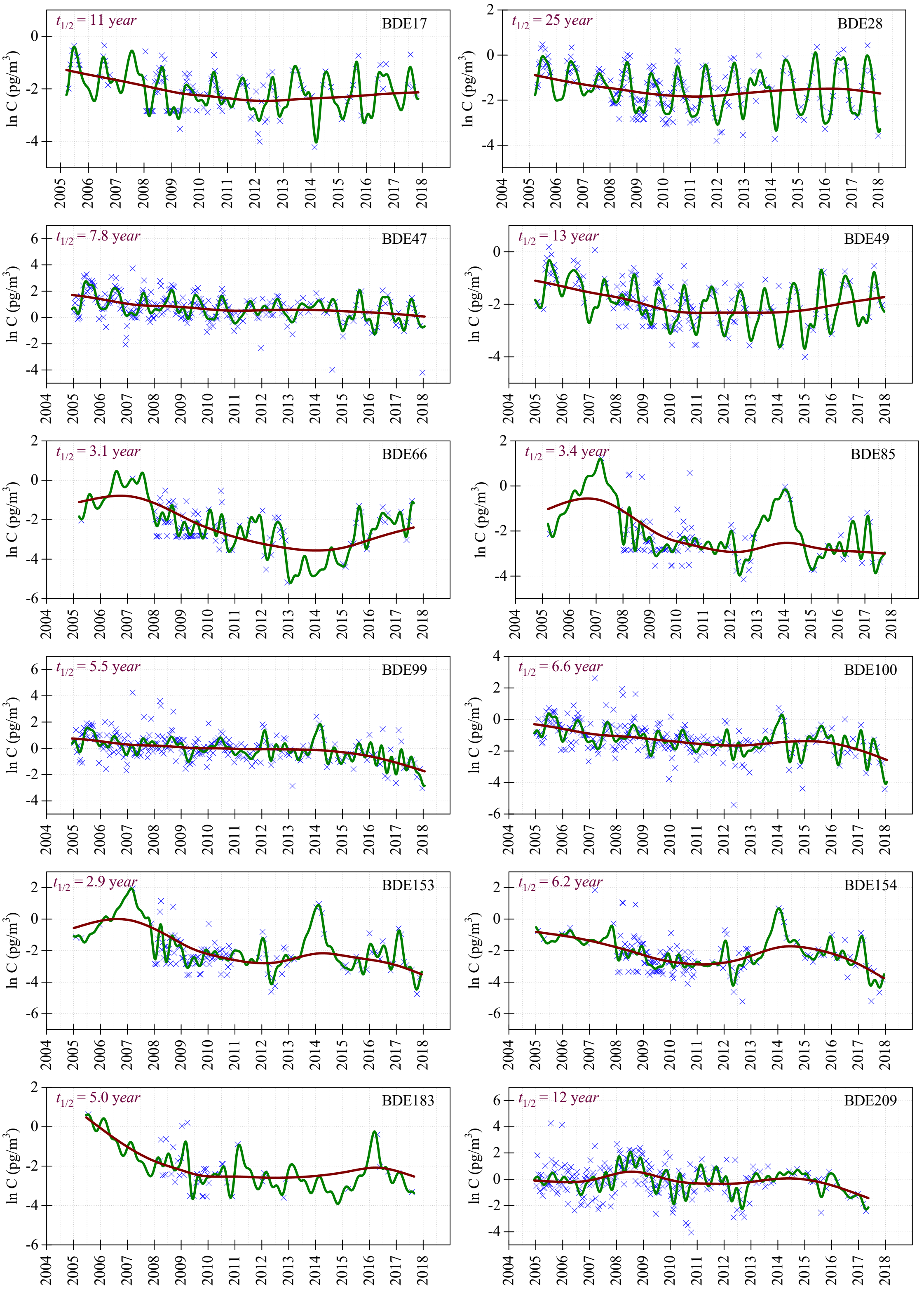

Figure S3. Trends of HFRs in air samples from Point Petre, Ontario, Canada.

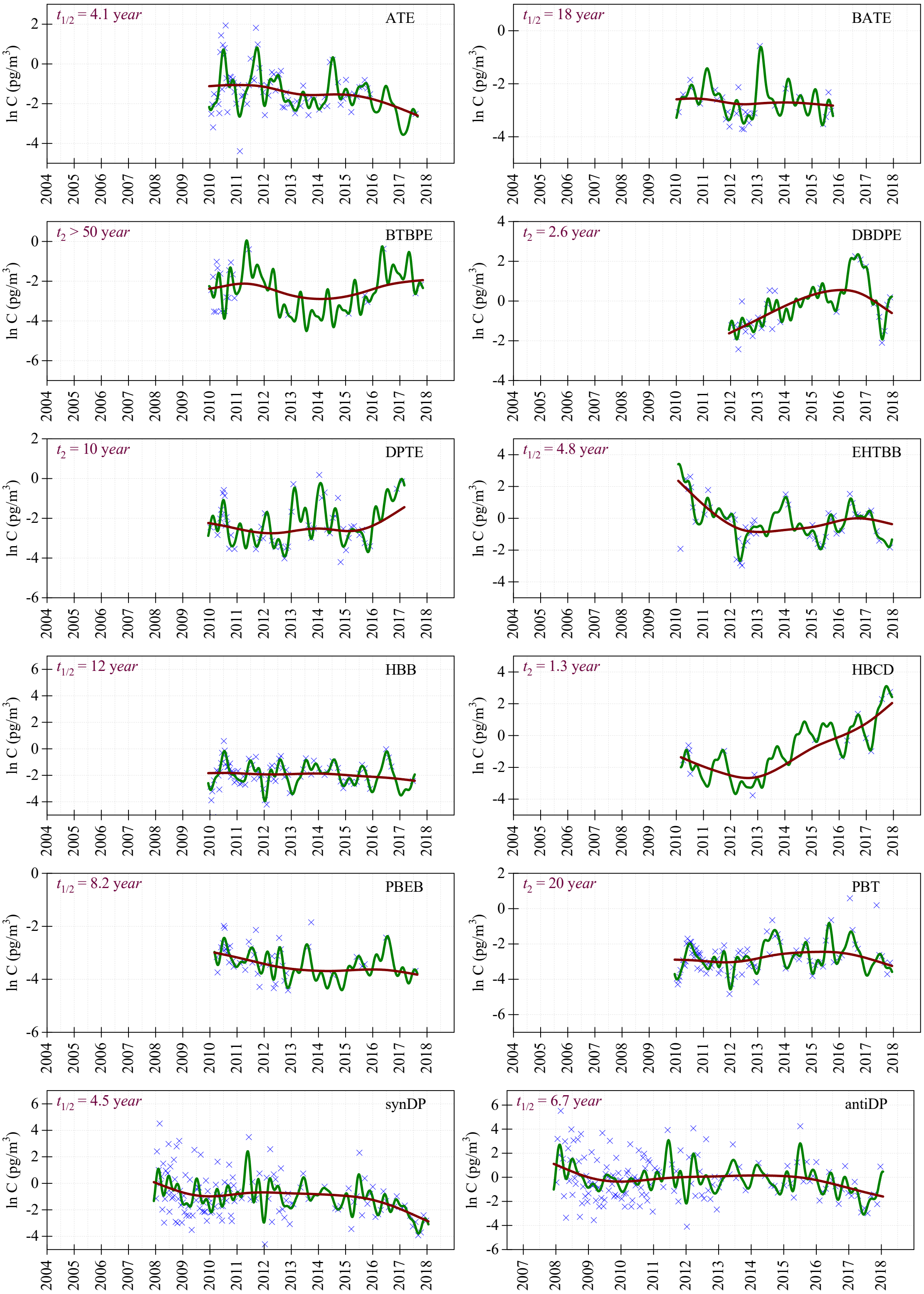

Figure S3 (Continued). Trends of HFRs in air samples from Point Petre, Ontario, Canada.

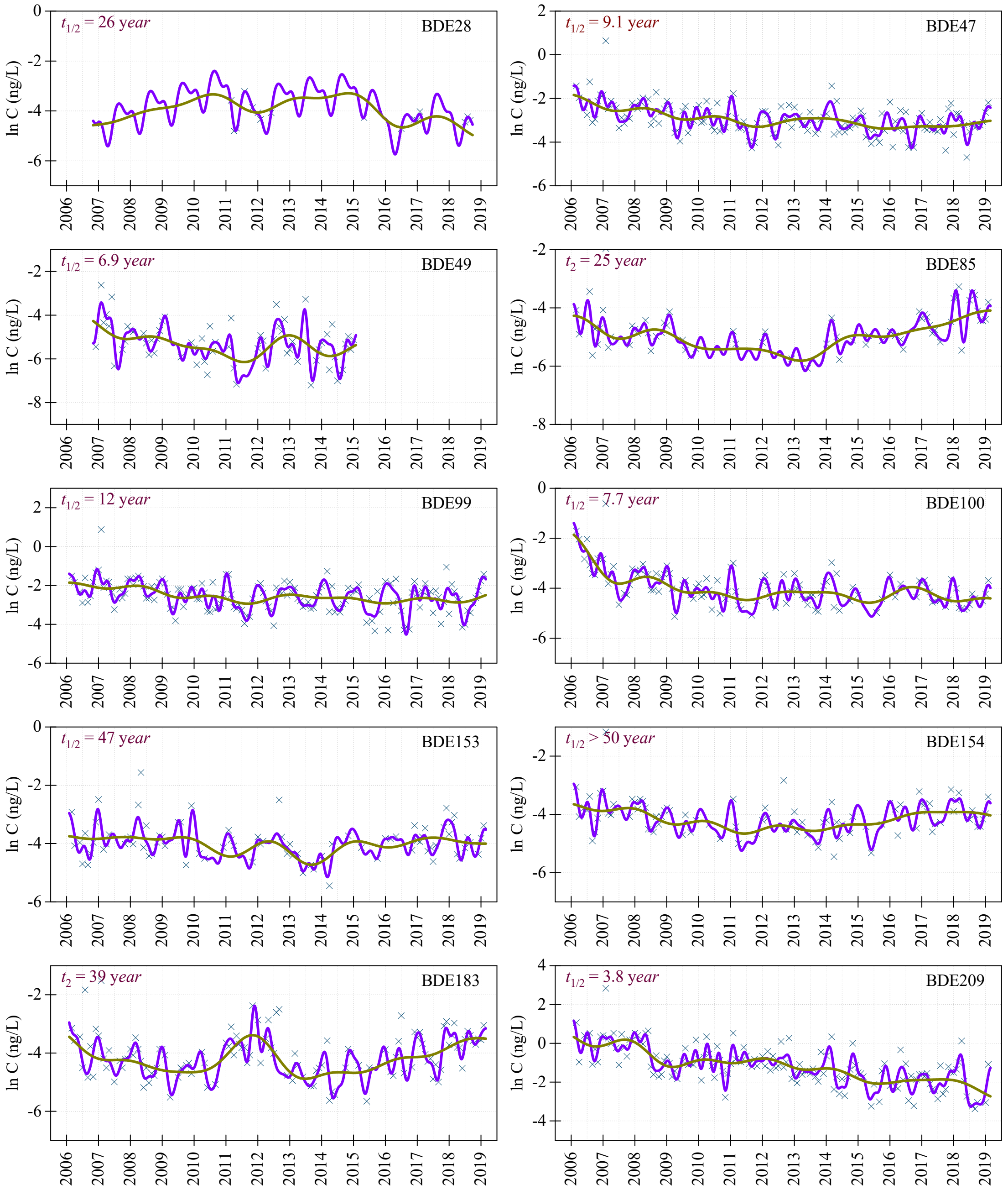

Figure S4. Trends of natural logarithm concentrations of HFRs in precipitation from Point Petre, Ontario, Canada.

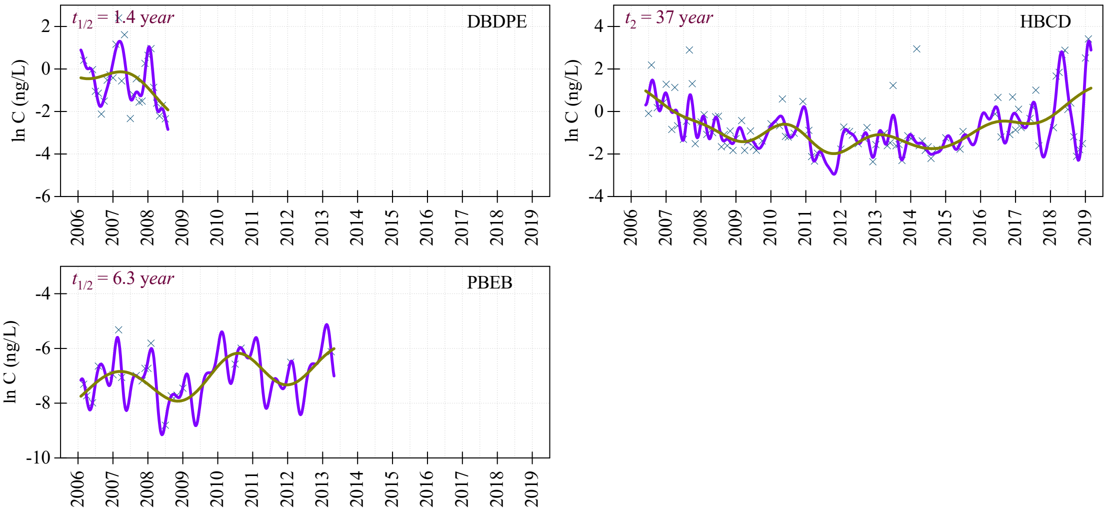

Figure S4 (Continued). Trends of HFR concentrations in precipitation from Point Petre, Ontario, Canada.

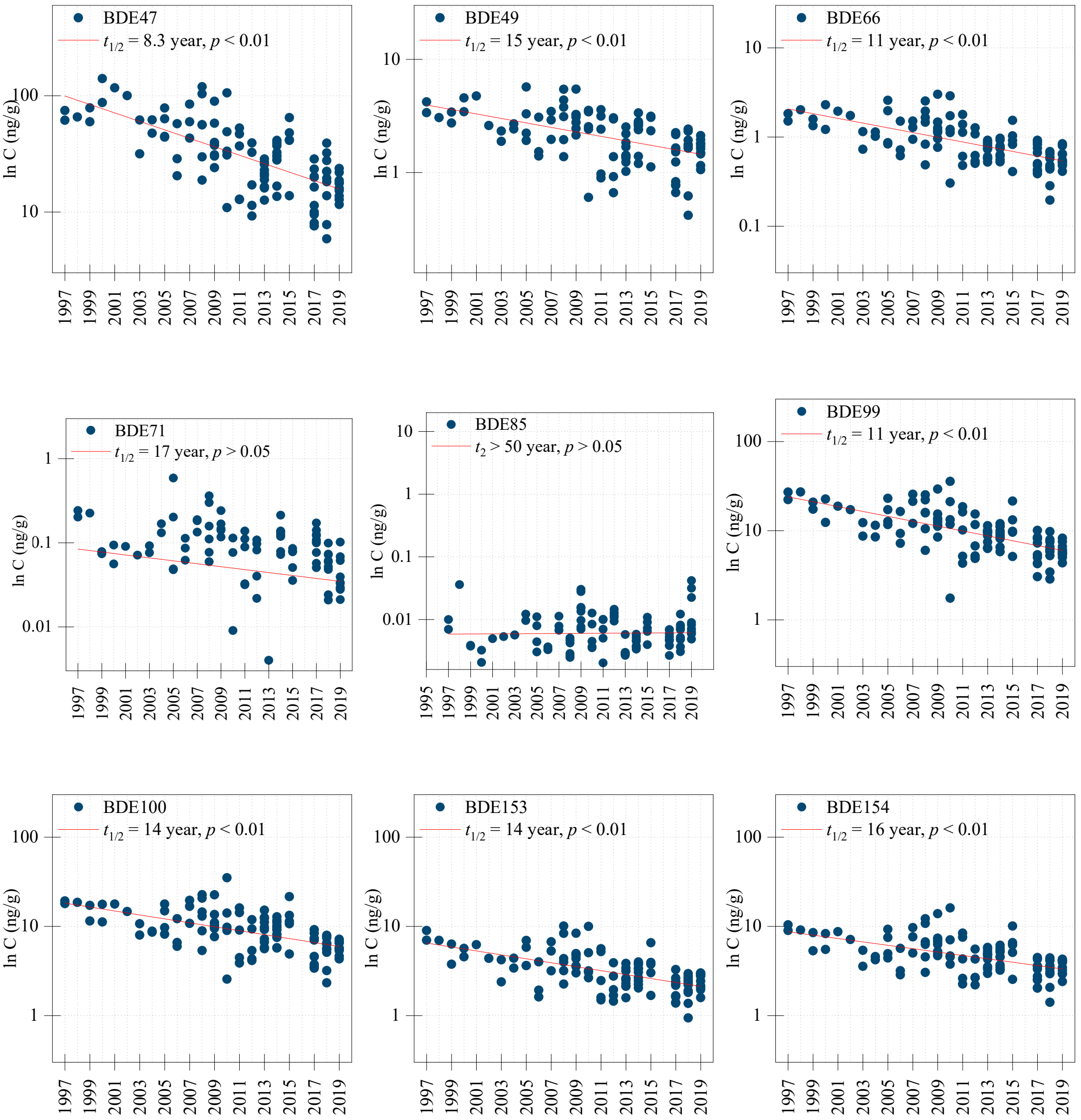

Figure S5. Trends of natural logarithm concentrations of HFRs in lake trout from Lake Ontario.

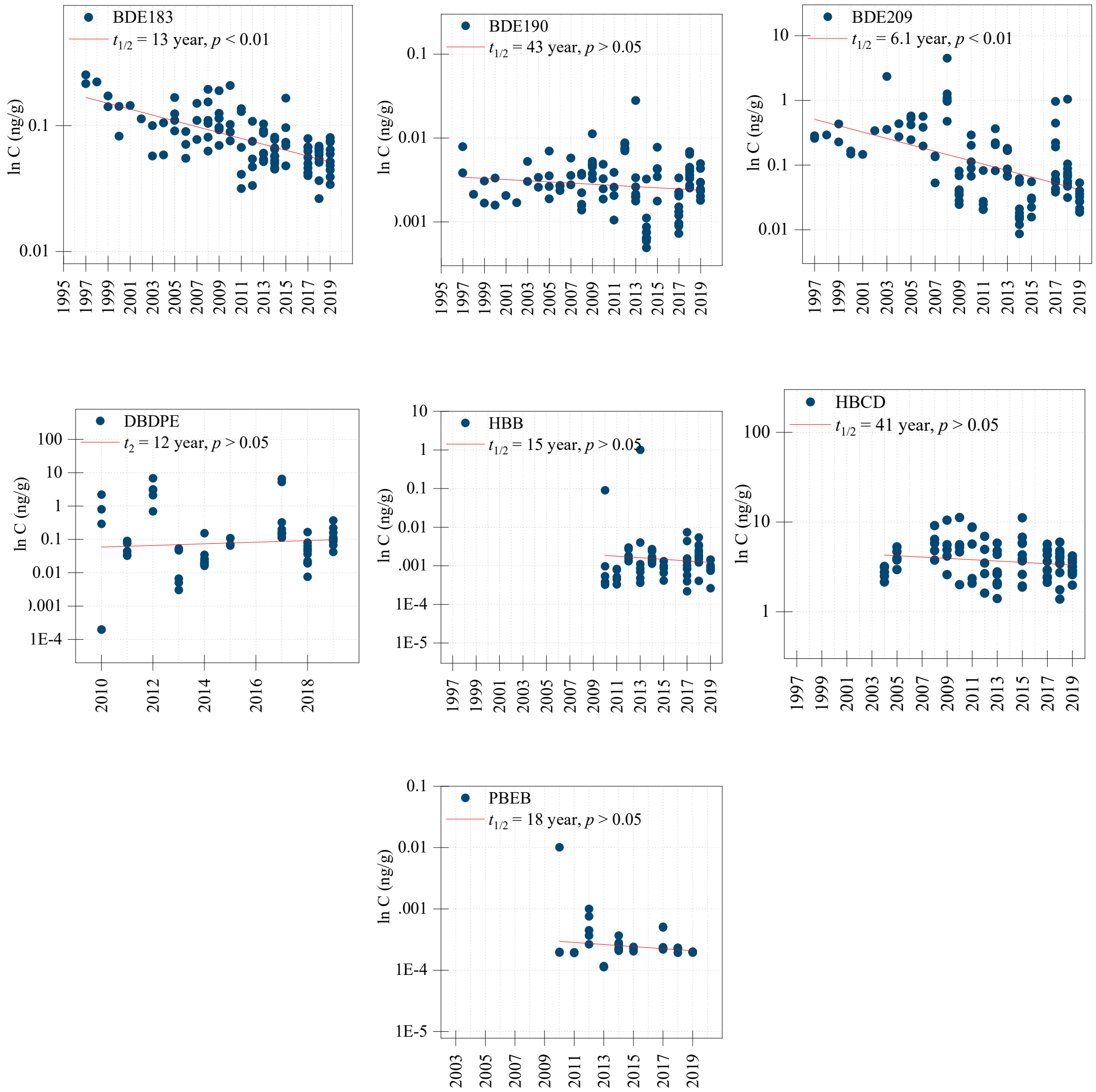

Figure S5 (Continued). Trends of HFR concentrations in lake trout from Lake Ontario.

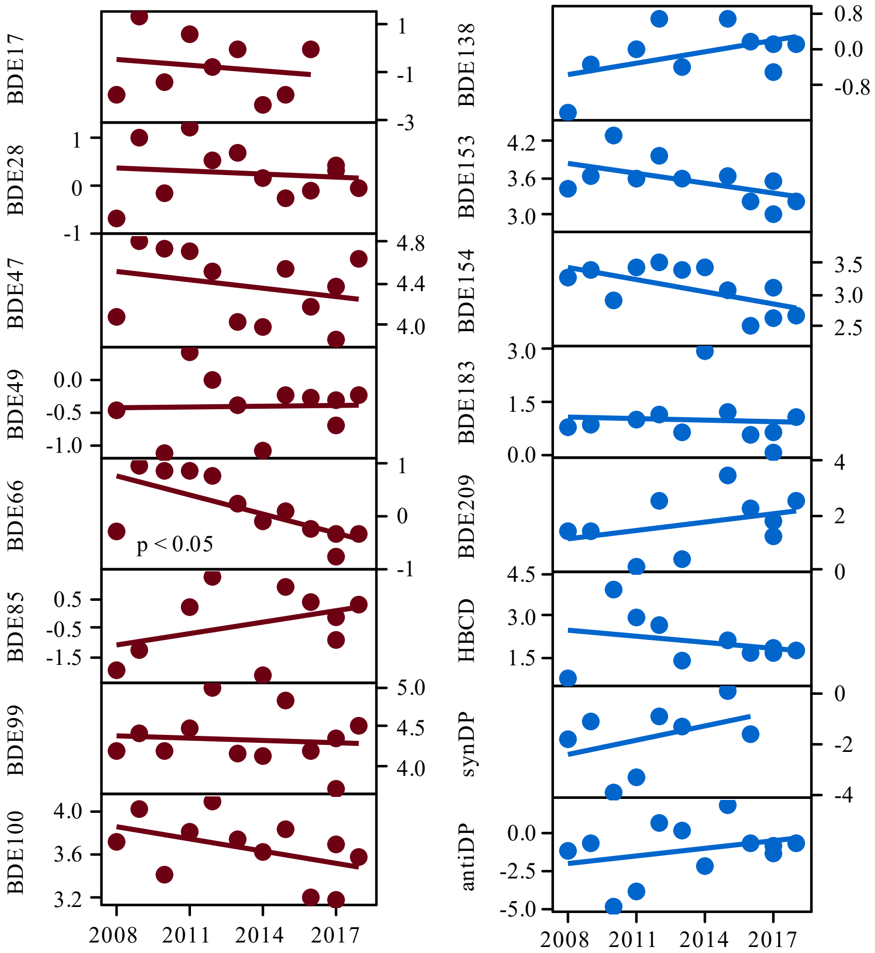

Figure S6. Trends of natural logarithm concentrations of HFRs in herring eggs from Lake Ontario.

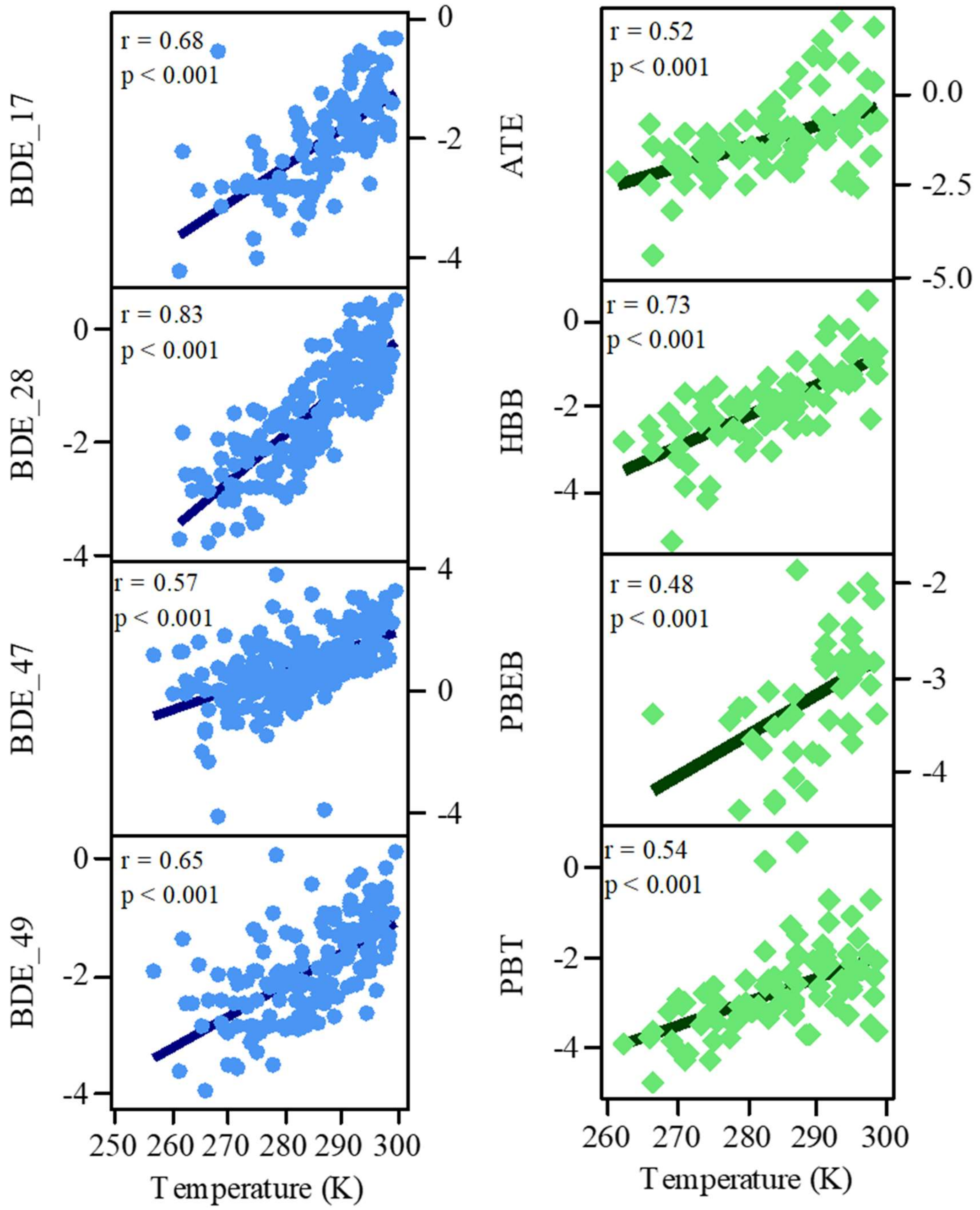

Figure S7. Correlations between HFR concentrations in the air and ambient temperature.

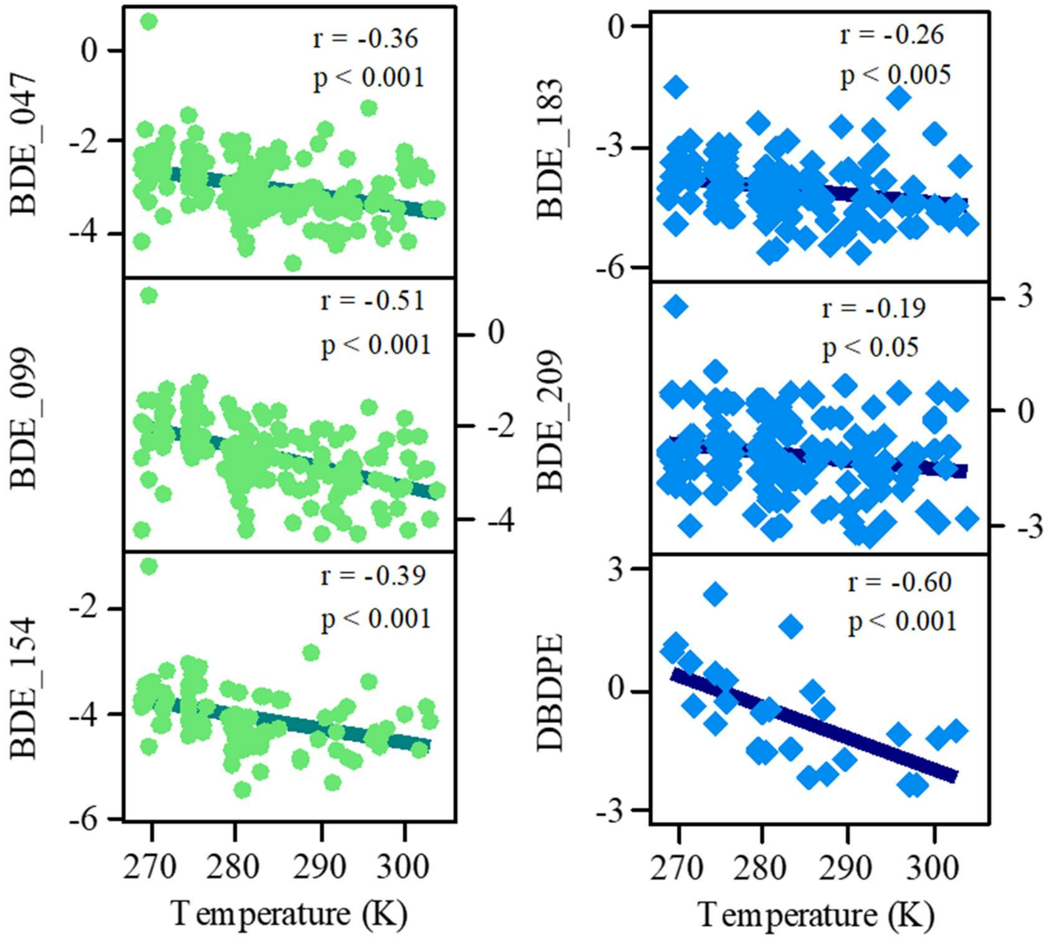

Figure S8. Correlation between the HFR concentrations in the precipitation and the ambient temperature.

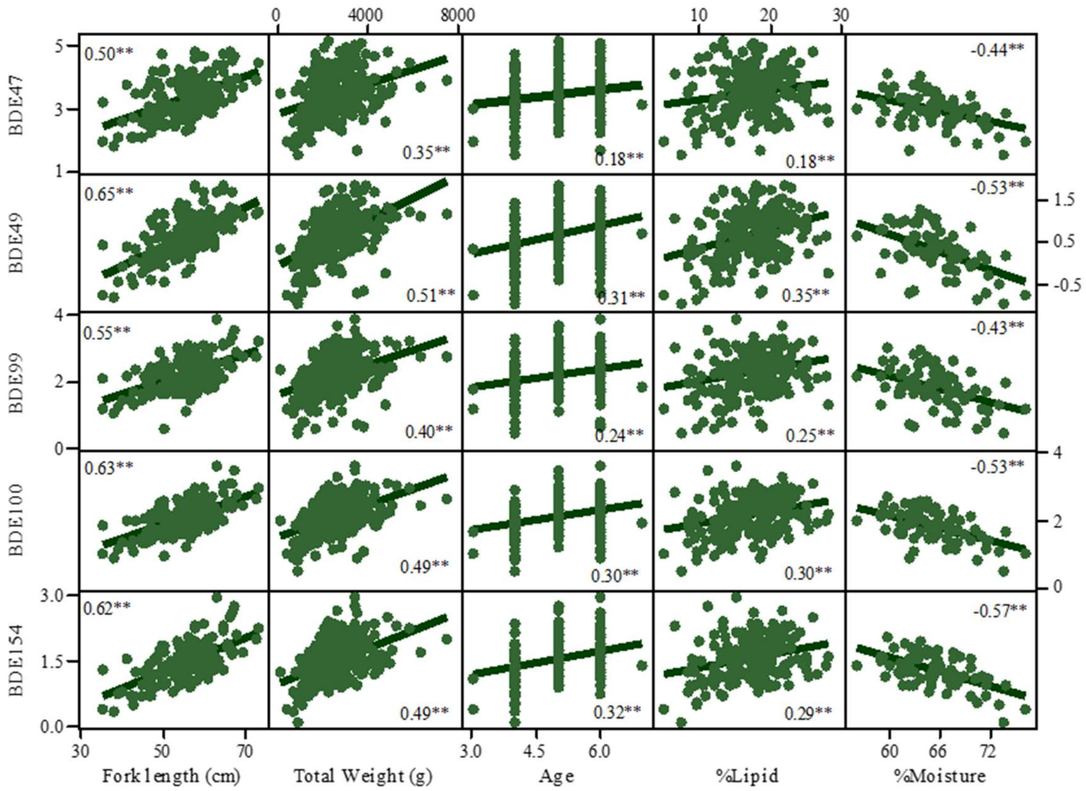

Figure S9. Lake trout HFR concentrations vs fork length, weight, age, lipid, and moisture.

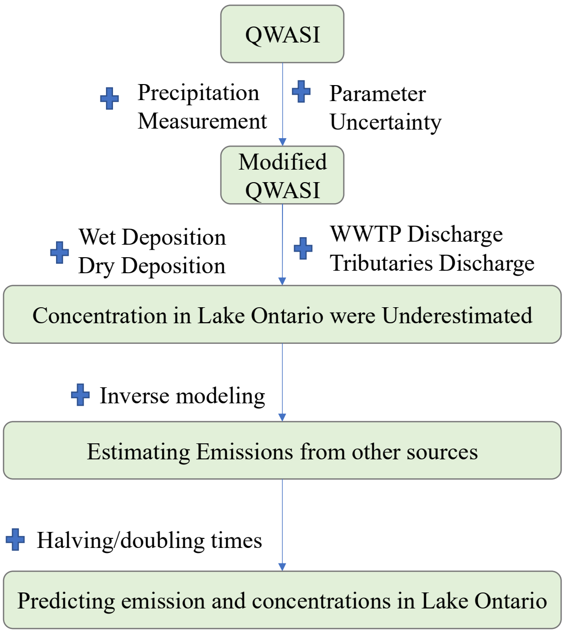

Figure S10. The workflow designed for implementing the modified QWASI involves estimating the temporal trends of HFR emissions and concentrations in water and sediment. Note that this method has been used in previous studies, such as evaluating officially reported polycyclic aromatic hydrocarbon emissions in the Athabasca oil sands region[11] and back-calculated the emissions of OPEs from the measured air concentrations.[12] While our study sought to investigate the temporal evolution of chemical contamination using the QWASI model, it is important to acknowledge that this approach assumes stable conditions over time. This assumption, though inherent to the steady-state model, may not fully capture scenarios with rapid changes and non-steady-state conditions, potentially affecting the accuracy of our model's predictions. As shown in **Table S8**, the calculated residence times for the water system were approximately 237 days, 242 days, and 86 days for BDE47, BDE209, and HBCD, respectively. Similarly, for the sediment system, the residence times were approximately 1330 days, 1330 days, and 630 days for BDE47, BDE209, and HBCD, respectively. In the water-sediment system, the calculated residence times were 858 days, 1130 days, and 148 days for BDE47, BDE209, and HBCD, respectively. Given these calculated residence times, we can conclude that the response time is not exceptionally long. Specifically, the water system is anticipated to reach a steady state within one year. These calculations provide us with valuable insights into the temporal dynamics of the model compartments, confirming that the timeframes involved do not hinder the potential for achieving a significant proportion of steady state within the studied timeframe. The timescales involved in sorption processes, which typically span months to years, could lead to differences in contaminant distribution between steady-state and non-steady-state scenarios.

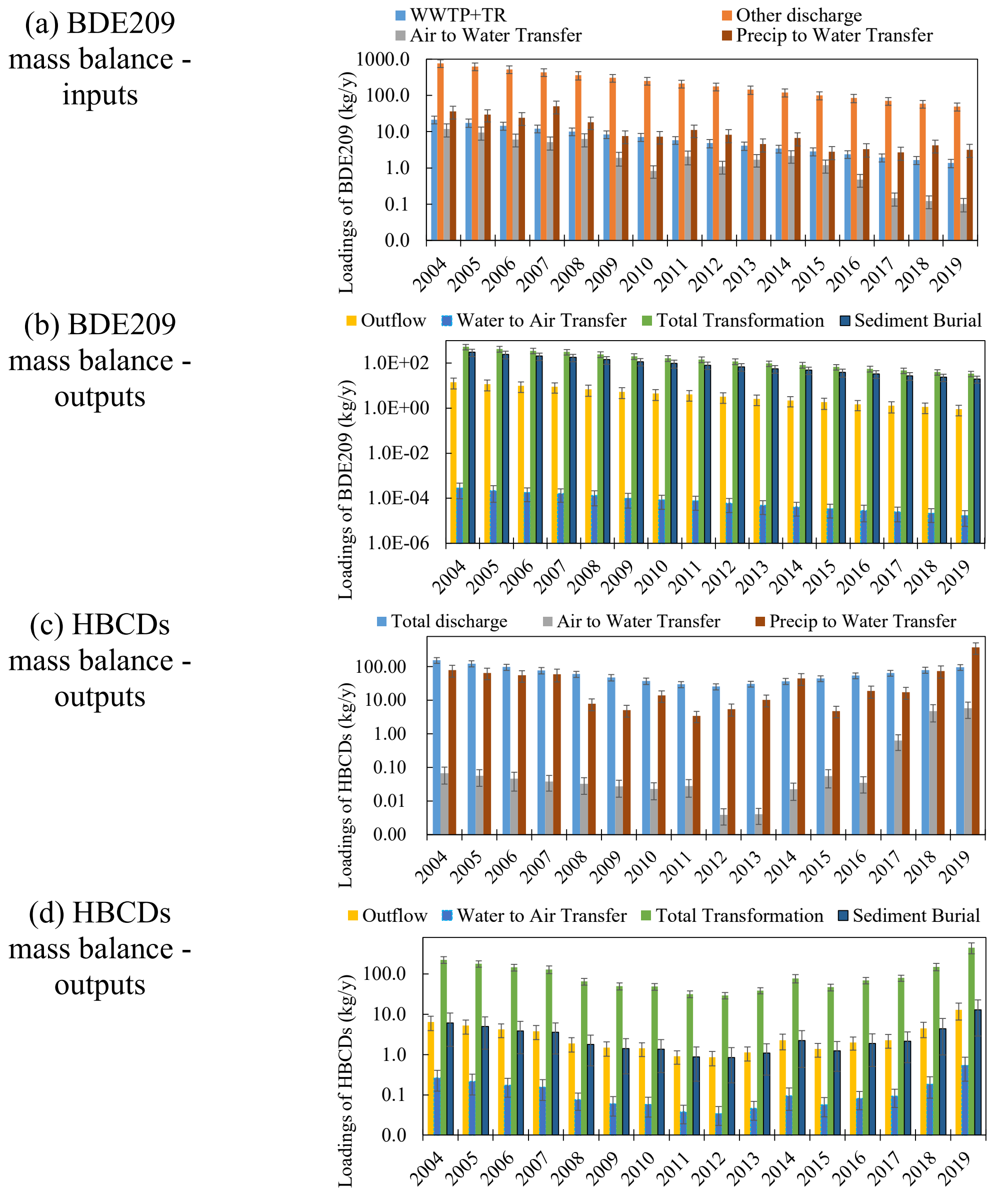

Figure S11. Trends of Transportation flux for (a, b) BDE209, and (c, d)HBCDs in Lake Ontario from 2004-2019.

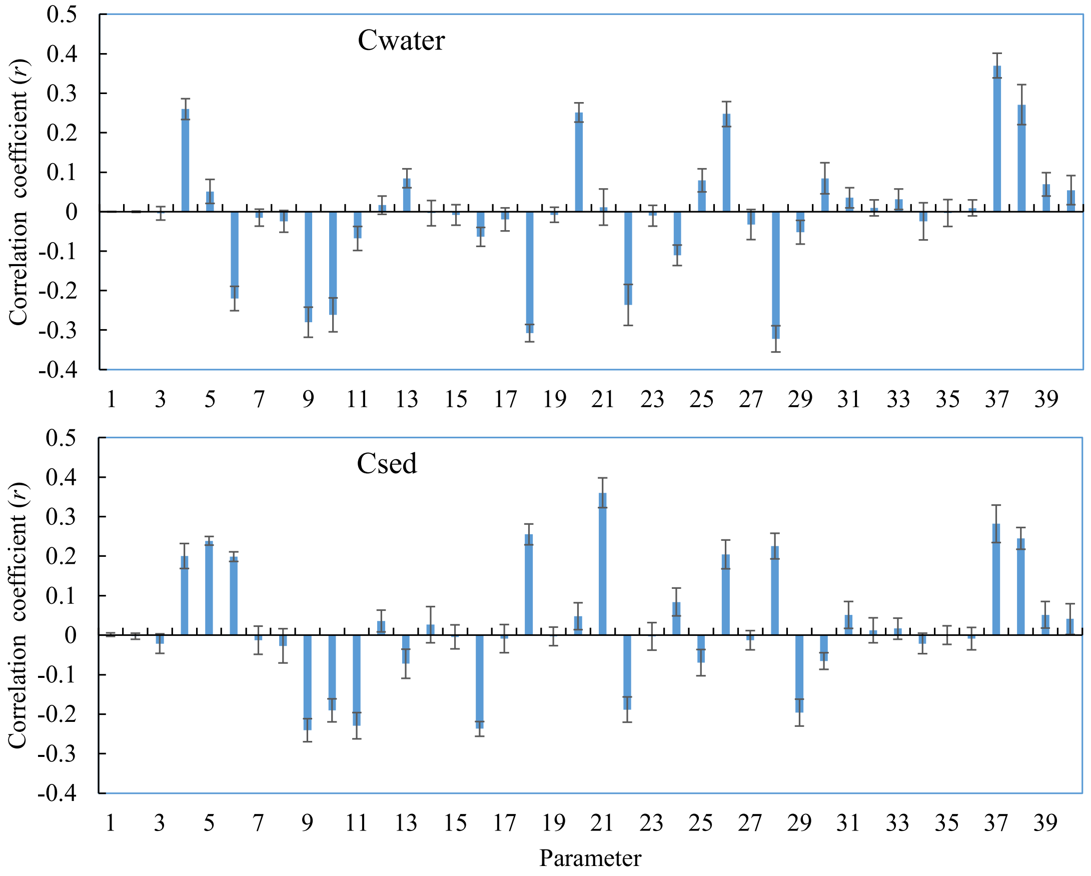

Figure S12. Sensitivity analyses on the parameters affecting the predicted concentrations of BDE47 in water (pg/L) and sediment (n/g dw).

Note: A higher absolute value of correlation coefficient (*r*) indicates a stronger relationship between the parameter being analyzed and the predicted concentrations.
These parameters are: 1: Air Temperature, 2: Water Temperature, 3: Henry's Law Constant, 4: Half-Lives in Water, 5: Half-Lives in Sediment, 6: K_{ow}, 7: K_{aw}, 8: Subcooled Liquid Vapor Pressure, 9: Water Surface Area, 10: Water Volume, 11: Sediment Active Layer Depth, 12: Aerosols in Air, 13: Solids in Water Column, 14: Solids in Inflow Water, 15: Solids in Precip, 16: Solids in Sediment, 17: Desity-Aerosol, 18: Desity-Particles in Warter, 19: Desity-Particles in Precipitation, 20: Desity-Sediment Solids, 21: Organic carbon (OC) in Particles in Lake Water, 22: OC in Particles in inflow, 23: OC in Particles in Precip, 24: OC in Sediment Solids, 25: OC in Resuspended Sediment, 26: Water Inflow, 27: Water Outflow, 28: Sedimentation, 29: Sediment Burial, 30: Sediment Resuspension, 31: Aerosol Deposition, 32: Scavenging Ratio, 33: Rain Rate, 34: mass transfer coefficient (MTC) Volatilization air side, 35: MTC Volatilization water side, 36: MTC Sediment-water Diffusion, 37: Directly Discharged into Water, 38: Concentration in Inflow (ng/L), 39: Concentration in Air (pg/m^3), and 40: Concentration in Precipitation (ng/L).

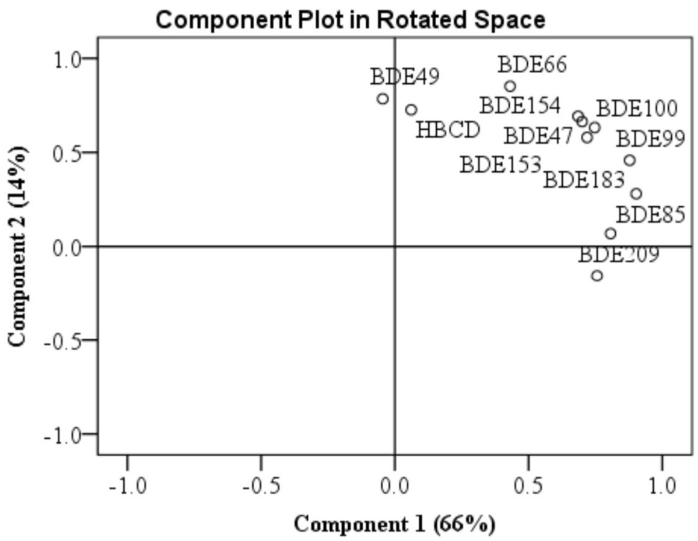

(a) Component plot

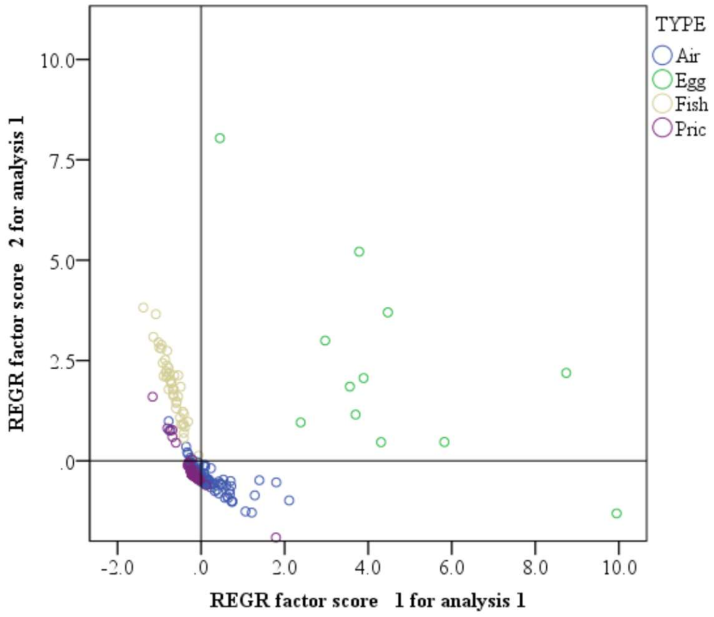

(b) Score plot

Figure S13. Principal component analysis (PCA) on selected HFRs in the air, precipitation (Pric), fish, and egg samples.

S5. References:

1. Shunthirasingham, C.; Alexandrou, N.; Brice, K. A.; Dryfhout-Clark, H.; Su, K.; Shin, C.; Park, R.; Pajda, A.; Noronha, R.; Hung, H. Temporal trends of halogenated flame retardants in the atmosphere of the Canadian Great Lakes Basin (2005-2014). *Environ. Sci. Process. Impacts* **2018,** *20*, (3), 469-479.
2. Shunthirasingham, C.; Gawor, A.; Hung, H.; Brice, K. A.; Su, K.; Alexandrou, N.; Dryfhout-Clark, H.; Backus, S.; Sverko, E.; Shin, C.; Park, R.; Noronha, R. Atmospheric concentrations and loadings of organochlorine pesticides and polychlorinated biphenyls in the Canadian Great Lakes Basin (GLB): Spatial and temporal analysis (1992-2012). *Environ. Pollut.* **2016,** *217*, 124-33.
3. Gewurtz, S. B.; Bradley, L. E.; Backus, S.; Dove, A.; McGoldrick, D.; Hung, H.; Dryfhout-Clark, H. Perfluoroalkyl Acids in Great Lakes Precipitation and Surface Water (2006-2018) Indicate Response to Phase-outs, Regulatory Action, and Variability in Fate and Transport Processes. *Environ. Sci. Technol.* **2019,** *53*, (15), 8543-8552.
4. McGoldrick, D. J.; Clark, M. G.; Keir, M. J.; Backus, S. M.; Malecki, M. M. Canada's national aquatic biological specimen bank and database. *J. Great Lakes Res.* **2010,** *36*, (2), 393-398.
5. Zhou, C.; Pagano, J.; McGoldrick, D. J.; Chen, D.; Crimmins, B. S.; Hopke, P. K.; Milligan, M. S.; Murphy, E. W.; Holsen, T. M. Legacy polybrominated diphenyl ethers (PBDEs) trends in top predator fish of the Laurentian Great Lakes (GL) from 1979 to 2016: will concentrations continue to decrease? *Environ. Sci. Technol.* **2019,** *53*, (12), 6650-6659.
6. McGoldrick, D. J.; Pelletier, M.; de Solla, S. R.; Marvin, C. H.; Martin, P. A. Legacy of legacies: Chlorinated naphthalenes in Lake Trout, Walleye, Herring Gull eggs and sediments from the Laurentian Great Lakes indicate possible resuspension during contaminated sediment remediation. *Sci. Total Environ.* **2018,** *634*, 1424-1434.
7. Melymuk, L.; Robson, M.; Csiszar, S. A.; Helm, P. A.; Kaltenecker, G.; Backus, S.; Bradley, L.; Gilbert, B.; Blanchard, P.; Jantunen, L.; Diamond, M. L. From the City

to the Lake: Loadings of PCBs, PBDEs, PAHs and PCMs from Toronto to Lake Ontario. *Environ. Sci. Technol.* **2014,** *48*, (7), 3732-3741.

8. Li, L.; Zhang, Z.; Men, Y.; Baskaran, S.; Sangion, A.; Wang, S.; Arnot, J. A.; Wania, F. Retrieval, selection, and evaluation of chemical property data for assessments of chemical emissions, fate, hazard, exposure, and risks. *ACS Environmental Au* **2022,** *2*, (5), 376-395.
9. Venier, M.; Dove, A.; Romanak, K.; Backus, S.; Hites, R. Flame Retardants and Legacy Chemicals in Great Lakes' Water. *Environ. Sci. Technol.* **2014,** *48*, (16), 9563-9572.
10. Yang, R. Q.; Wei, H.; Guo, J. H.; Li, A. Emerging Brominated Flame Retardants in the Sediment of the Great Lakes. *Environ. Sci. Technol.* **2012,** *46*, (6), 3119-3126.
11. Parajulee, A.; Wania, F. Evaluating officially reported polycyclic aromatic hydrocarbon emissions in the Athabasca oil sands region with a multimedia fate model. *Proc. Natl. Acad. Sci. U. S. A.* **2014,** *111*, (9), 3344-3349.
12. Rodgers, T. F.; Giang, A.; Diamond, M. L.; Gillies, E.; Saini, A. Emissions and fate of organophosphate esters in outdoor urban environments. *Nature communications* **2023,** *14*, (1), 1175.
